# Drug Repurposing Approach to Identify Candidate Drug Molecules for Hepatocellular Carcinoma

**DOI:** 10.3390/ijms25179392

**Published:** 2024-08-29

**Authors:** Tugce Baser, Ahmet Sureyya Rifaioglu, Mehmet Volkan Atalay, Rengul Cetin Atalay

**Affiliations:** 1 Department of Health Informatics, Graduate School of Informatics, Middle East Technical University, 06800 Ankara, Türkiye; 2 Institute for Computational Biomedicine, Faculty of Medicine, Heidelberg University Hospital, Heidelberg University, Bioquant, 69117 Heidelberg, Germany; 3 Department of Electrical and Electronics Engineering, Faculty of Engineering, İskenderun Technical University, 31200 Hatay, Türkiye; 4 Department of Computer Engineering, Faculty of Engineering, Middle East Technical University, 06800 Ankara, Türkiye

**Keywords:** drug candidate, drug repurposing, hepatocellular carcinoma, machine learning, MDeePred

## Abstract

Hepatocellular carcinoma (HCC) is the most prevalent primary liver cancer, with a high mortality rate due to the limited therapeutic options. Systemic drug treatments improve the patient’s life expectancy by only a few months. Furthermore, the development of novel small molecule chemotherapeutics is time-consuming and costly. Drug repurposing has been a successful strategy for identifying and utilizing new therapeutic options for diseases with limited treatment options. This study aims to identify candidate drug molecules for HCC treatment through repurposing existing compounds, leveraging the machine learning tool MDeePred. The Open Targets Platform, UniProt, ChEMBL, and Expasy databases were used to create a dataset for drug target interaction (DTI) predictions by MDeePred. Enrichment analyses of DTIs were conducted, leading to the selection of 6 out of 380 DTIs identified by MDeePred for further analyses. The physicochemical properties, lipophilicity, water solubility, drug-likeness, and medicinal chemistry properties of the candidate compounds and approved drugs for advanced stage HCC (lenvatinib, regorafenib, and sorafenib) were analyzed in detail. Drug candidates exhibited drug-like properties and demonstrated significant target docking properties. Our findings indicated the binding efficacy of the selected drug compounds to their designated targets associated with HCC. In conclusion, we identified small molecules that can be further exploited experimentally in HCC therapeutics. Our study also demonstrated the use of the MDeePred deep learning tool in in silico drug repurposing efforts for cancer therapeutics.

## 1. Introduction

Cancer is a significant global health burden, standing as the primary reason for early mortality (specifically between ages 30 and 69) in 134 out of 183 nations and holding the third or fourth position in another 45 countries [1]. If current global trends persist, it is projected that the worldwide cancer cases will surge by over 60%, reaching approximately 29.4 million cases by 2040 [2].

The primary liver cancer, hepatocellular carcinoma (HCC), arises due to chronic liver conditions, frequently resulting from infections like hepatitis B or C, excessive alcohol consumption, or metabolic disorders [3]. Primary liver cancer ranks among the most prevalent cancers across the globe, with an increasing incidence in Western nations due to obesity-associated chronic liver disease [4]. Globally, its death-to-diagnosis ratio stands at 0.91. It is diagnosed 2.3 times more in men compared to women, and a significant 72% of these new cases emerge in Asia [5].

Currently, recommended curative treatments for HCC include surgical resection, liver transplantation (LT), and radiofrequency ablation (RFA) [6]. However, only one-third of HCC patients are eligible for these curative methods. The majority of the patients undergo non-curative treatments like transarterial chemoembolization (TACE) and systemic medications, such as molecular targeted agents (MTAs), monoclonal antibodies, or immune checkpoint inhibitors, as their primary treatment [7]. Finally, advanced-stage tumors are typically treated with systemic medications like combinations such as atezolizumab and bevacizumab, as well as individual drugs like sorafenib, lenvatinib, regorafenib, cabozantinib, and ramucirumab [8].

For treatment options for cancer in general, including primary liver cancer, targeted drug therapies stand out as effective methods. Pre-clinical and clinical studies, manufacturing procedures, target identification and validation, therapeutic screening, and lead compound optimization are complex and protracted processes involved in the systemic drug development process. All of these stages present significant difficulties in the quest to identify efficacious systemic drugs for tackling cancer [9]. The current systemic drug discovery process is not only costly but also time-inefficient. Introducing a new drug to the market requires about 15 years of time, irrespective of the cost [10]. Given the prolonged nature of drug development, drug repurposing has emerged as a beneficial and effective approach to discovering and developing new drug molecules. It is highlighted as a method that saves both time and money in delivering new therapeutic agents. Since the safety, dosage, and toxicity of existing drugs are typically already established, they can move through clinical stages faster than brand-new drugs [11]. 

In light of these challenges and considerations, this study aims to identify candidate drug molecules for HCC therapeutics through drug repurposing among existing compounds found in small molecule databases. To achieve this, we employed a recently developed deep learning-based method named MDeePred [12]. Our proposed approach holds promise in streamlining the drug discovery process by leveraging existing data and computational methods to identify potential therapeutic options not limited to HCC but also for other cancers.

## 2. Results

### 2.1. Datasets

To find HCC-related genes, the Open Targets Platform database was used. The Open Targets Platform integrates an extensive genetic and biomedical database to comprehensively analyze genes associated with complex diseases. Utilizing this platform to identify genes responsible for HCC is crucial for better understanding the genetic basis of the disease and pinpointing specific genes and mutations that contribute to its development. The Open Targets Platform enables genetic associations, somatic mutations, biological pathways, and their connections to a disease such as HCC. This facilitates more accurate identification of genes directly involved in the pathogenesis of HCC. By using this platform, we identified high-confidence 7853 HCC-related data, which is a key step towards identifying potential therapeutic targets and developing new treatment approaches for HCC. After genetic associations and somatic mutations selection, 673 genes were obtained that were associated with HCC (Table S1). The selection of “genetic associations and somatic mutations” was crucial in the identification of genes directly associated with HCC. Genetic associations reveal disease-linked variants and their potential roles in the development of HCC, while somatic mutations identify genetic changes that occur within cancer cells, directly contributing to tumor formation. By combining these two criteria, the selection of HCC-associated genes becomes more specific and targeted for therapeutics. Therefore, using these two approaches together not only identifies HCC-associated genes but also enhances our understanding of their impact on HCC. This step is vital for determining potential biomarkers and therapeutic targets, thereby increasing the accuracy and relevance of the research.

To obtain the set of genes used in this research, the arithmetic mean of the genetic associations and somatic mutation scores was calculated. Then, genes that have an arithmetic mean value 0.25 and above were selected. The cut-off value of 0.25 was chosen to filter genes strongly associated with HCC, ensuring only the most relevant genes are selected while minimizing false positives. This threshold balances the gene pool, avoiding excessive noise without excluding significant candidates. By combining genetic association and somatic mutation data, this cut-off enhances the reliability of the study and ensures biologically meaningful results. Thus, 106 genes that are associated with HCC were selected as our actual genes. Table S2 shows 106 genes and their genetic associations and somatic mutation scores. 

By using the ChEMBL database, a compound–protein activity training dataset for each target was created. While choosing compounds, the IC_50_ and pChEMBL values were taken into account, and some filtering criteria were done (refer to Table S3). After that, 46,400 data (for 106 genes) were grouped to select the transferases by using the ChEMBL, UniProt, and Expasy databases. At first, it was checked whether the targets were enzymes or not. Then, the enzyme class of the targets that were enzymes were determined. Finally, 22 targets (38,794 data) were identified as transferases (Table 1). 

The genes given in Table 1 play critical roles in the pathogenesis of HCC, affecting tumor cell survival, proliferation, and metastasis. *TERT* and *PIK3CA* are involved in telomerase activity and the PI3K/AKT signaling pathway, promoting cell immortalization and survival [13]. Tyrosine kinase receptors such as *MET*, *ALK*, *RET*, and *ROS1* facilitate HCC cell invasion and metastasis by regulating growth, migration, and differentiation [14]. Epigenetic regulators like *CREBBP* and *SETD2* control gene expression through histone modifications, with their dysregulation leading to tumor development [15,16,17]. Signaling pathways involving *AKT1* and *MAP3K1* influence cell survival and apoptosis, enhancing tumor growth when activated [18,19,20,21,22]. VEGF receptors (*KDR*, *FLT4*, *FGFR1*, and *FLT3*) and FGF receptors support tumor angiogenesis, providing essential nutrients for growth [23,24]. *KIT* and *ERBB3* receptors regulate cellular growth and differentiation, driving tumor proliferation [25,26,27]. *JAK3* and *NTRK1* impact signaling pathways and immune responses, while *ATM* and *KMT2A* are involved in DNA repair and gene expression [28,29,30,31,32]. Protein kinases such as *ACVR2A* and *PRKACA* participate in signaling pathways governing cell growth and differentiation, with their dysregulation contributing to tumor progression [33,34,35]. Given their crucial roles in HCC, these genes are valuable therapeutic targets, and their inhibitors or modulators hold potential to halt or slow disease progression [36].

### 2.2. Results of MDeePred

The MDeePred technique was selected as the machine learning approach to identify potential drug candidates for HCC. To create the train and test datasets, data with more than one datum of the same gene for the same molecule, coming from different experiments, were deduplicated. To handle the duplicate data, we used the median bioactivity value. As a result, a total of 38,794 data for 22 tranferases was reduced to 30,821 data. The train and test datasets were created by using deduplicated data. After that, MDeePred was performed with these datasets. We obtained 380 DTIs after MDeePred (Table S4).

Six DTIs, which target–compound relationship has been studied in the literature, among 380 DTIs were decided to be used for further studies (Table 2).

### 2.3. Enrichment Analyses of the MDeePred Results

**Figure 1:**
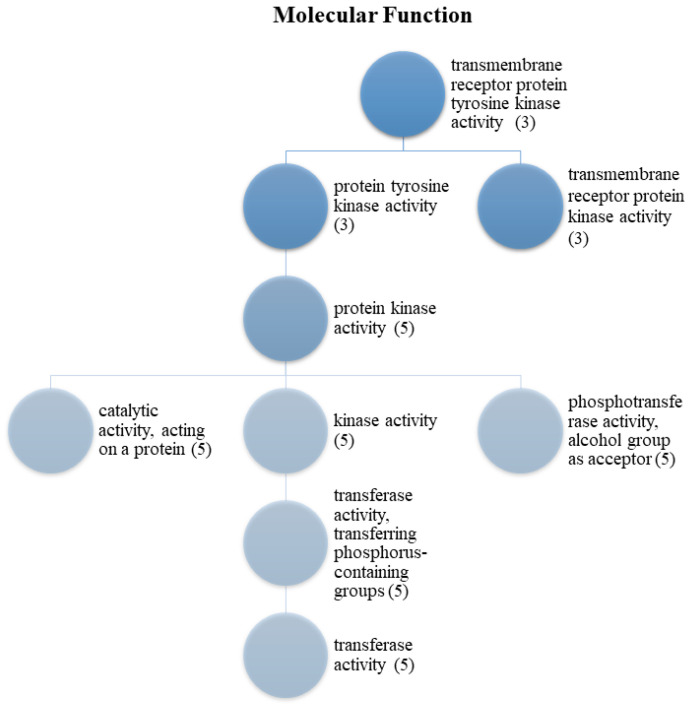
Transmembrane receptor protein tyrosine kinase activity of the protein set. (**3**) FGFR1, ALK, and FLT3; (**5**) FGFR1, ALK, AKT1, FLT3, and PIK3CA.

**Figure 2:**
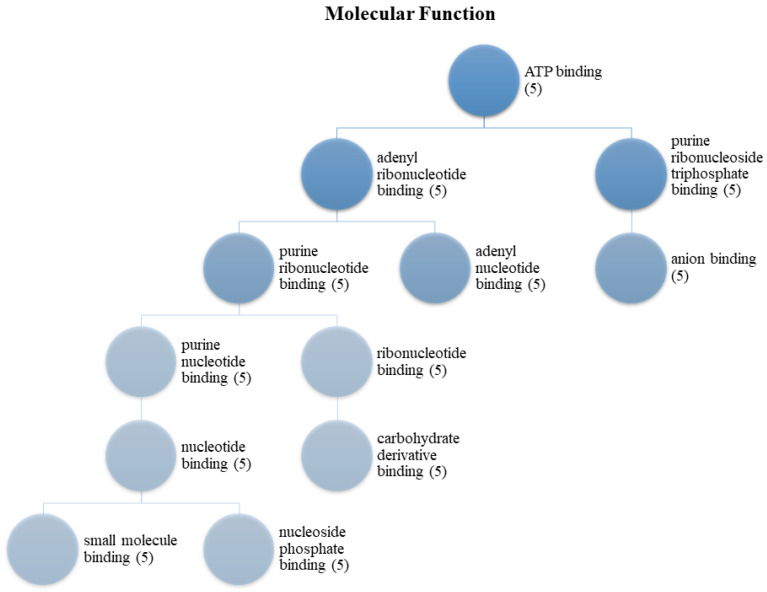
ATP binding of the protein set. (**5**) FGFR1, ALK, AKT1, FLT3, and PIK3CA.

**Figure 3:**
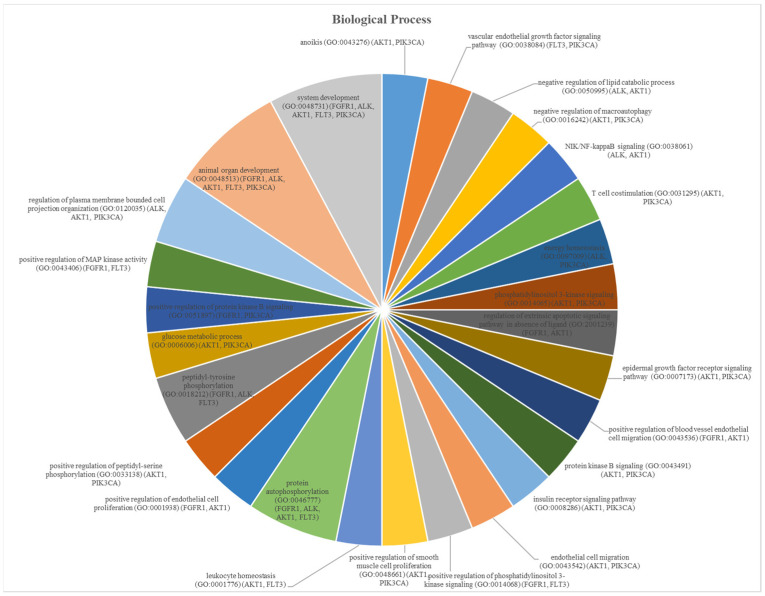
Biological process analyses of the protein set.

For HCC, molecular function enrichment analysis is crucial for the identification of therapeutic targets, understanding the disease mechanism, diagnosis and prognosis of the disease, and evaluating the treatment response. Therefore, molecular function enrichment analysis for HCC is a critical tool to understand the molecular basis of this cancer, potential treatment targets, and treatment responses. As a result of enrichment analyses of the MDeePred results, molecular functions were grouped into two categories. These are transmembrane receptor protein tyrosine kinase activity (Figure 1) and ATP binding (Figure 2). Biological process analyses were grouped into 27 main categories (Figure 3).

### 2.4. SwissADME and Molecular Docking Results

SwissADME and molecular docking are critically important tools for evaluating, optimizing, and selecting potential drug candidates for HCC treatment. These tools can expedite the drug development process, contributing to the development of more effective and safer treatments.

**Figure 4:**
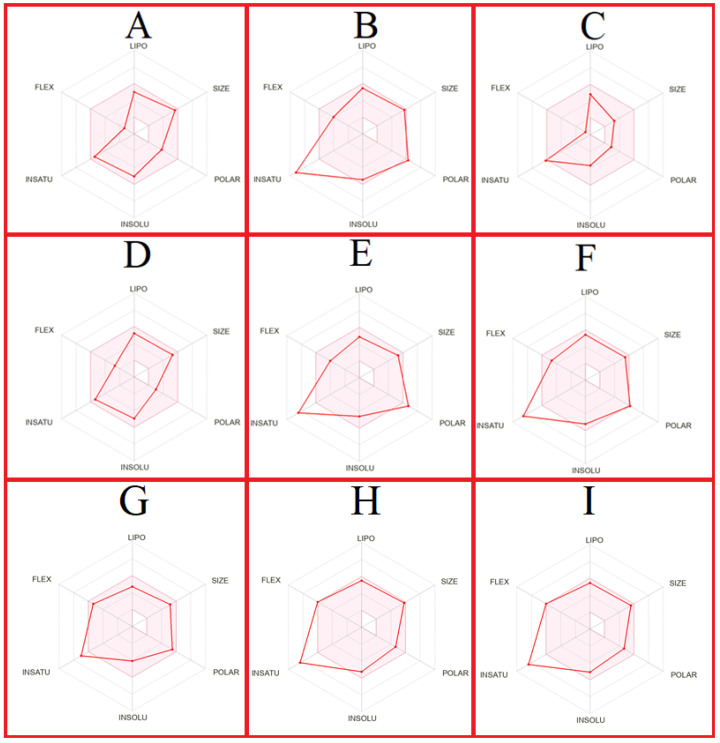
Schematic diagram of the oral bioavailability of the drug candidate compounds and drugs. (**A**) CHEMBL388978. (**B**) CHEMBL1615189. (**C**) CHEMBL328029. (**D**) CHEMBL1165499. (**E**) CHEMBL1773581. (**F**) CHEMBL1773601. (**G**) Lenvatinib. (**H**) Regorafenib. (**I**) Sorafenib.

The schematic diagram of oral bioavailability is used to quickly assess the pharmacokinetic properties (lipophilicity, size, polarity, insolubility, insaturation, and flexibility) of a drug candidate. This is particularly important in the drug design and development stage to predict the oral bioavailability of potential drug candidates. A schematic diagram of the oral bioavailability of the drug candidate compounds, lenvatinib, regorafenib, and sorafenib is illustrated in Figure 4. 

**Figure 5:**
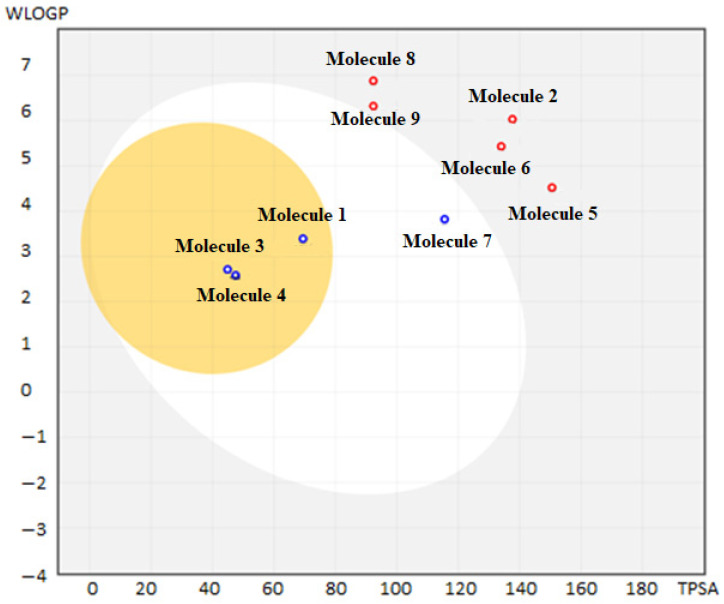
BOILED-Egg diagram of the drug candidate compounds and drugs. (**1**) CHEMBL388978. (**2**) CHEMBL1615189. (**3**) CHEMBL328029. (**4**) CHEMBL1165499. (**5**) CHEMBL1773581. (**6**) CHEMBL1773601. (**7**) Lenvatinib. (**8**) Regorafenib. (**9**) Sorafenib.

The BOILED-Egg diagram is a graphical tool used to predict a molecule’s overall absorption, distribution, metabolism, and excretion (ADME) properties. The BOILED-Egg represents predictions on the gastrointestinal absorption (GIA) and the ability of a molecule to cross the blood–brain barrier (BBB). The BOILED-Egg diagram of the drug candidate compounds and approved HCC drugs is illustrated in Figure 5.

The predictive findings related to physicochemical characteristics, lipophilicity, water solubility, pharmacokinetics, drug-likeness, and medicinal chemistry of the drug candidate compounds, lenvatinib, regorafenib, and sorafenib are illustrated in Table 3 and Tables S5–S8.

**Figure 6:**
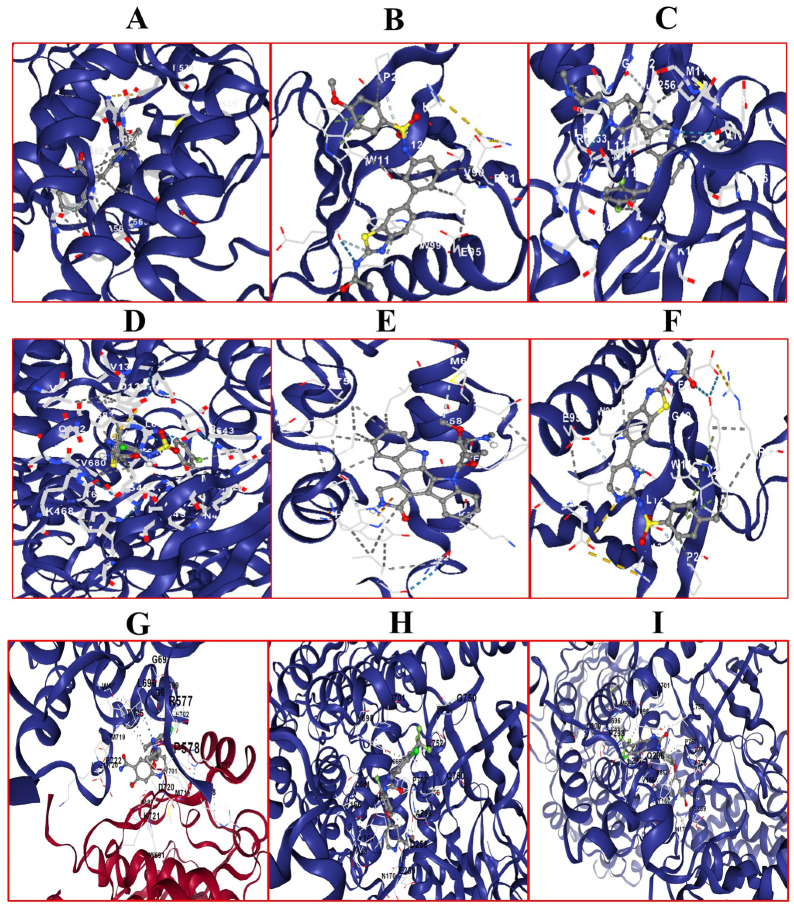
The best poses in the molecular docking of DTIs. (**A**) FGFR1 and CHEMBL328029. (**B**) ALK and CHEMBL1165499. (**C**) AKT1 and CHEMBL1773601. (**D**) AKT1 and CHEMBL1773581. (**E**) FLT3 and CHEMBL388978. (**F**) PIK3CA and CHEMBL1615189. (**G**) FGRF1 and levatinib. (**H**) PIK3CA and regorafenib. (**I**) PIK3CA and sorafenib. Blue dotted lines for hydrogen bonds, yellow for electrostatic interactions, and grey for hydrophobic interactions allow to observe how our drug compounds interact with target proteins.

In addition, molecular docking analyses were applied to the selected six DTIs, which contain five different protein targets from the transferase class after the MDeePred analysis, six drug candidates together with lenvatinib, regorafenib, and sorafenib. In Figure 6, drug candidates and drugs for human HCC are illustrated, along with the best poses in their docking with the binding site of their targets.

Additionaly, Table 4 shows the docking results (vina score, cavity volume (Å^3^), and contact residues) of lenvatinib, regorafenib, and sorafenib and our six DTI transferases. 

### 2.5. Literature-Based Validation of Novel DTI Predictions towards Drug Repurposing

As a result of the literature review of 380 DTIs, for only 6 DTIs, publications showing the target compound relationship were found. Table 5 lists the DTI predictions for each interaction that have been supported by the literature, along with the original source. In addition, the IC_50_ values of six DTIs were obtained from ChEMBL.

## 3. Discussion

HCC is a common malignant tumor in the digestive system. It ranks fifth in incidence and third in fatality rate among all malignant tumors globally. Primary liver cancer often develops without noticeable symptoms, and the majority of cases are diagnosed at an intermediate or advanced stage, resulting in a poor prognosis [41]. While systemic chemotherapy has improved survival rates in HCC patients, progress in treatment outcomes remains slow and insufficient [42]. Additionally, the development of new drugs is both a lengthy and expensive process that typically takes 10–15 years to develop a new drug candidate, with an average success rate of only 2.01% [43]. Drug repurposing leverages approved or investigational drugs for applications beyond their original medical indications. The main advantage is that their pharmacokinetic, pharmacodynamic, and toxicity profiles are already established from early studies. This allows these drugs to quickly progress to phase II and III clinical trials [44].

In this study, we aimed to identify candidate therapeutic compounds for HCC by repurposing existing small molecule drugs using a machine learning approach named MDeePred. MDeePred was used to identify potential drug candidates targeting genes responsible for HCC through a DTI study. We identified 380 DTIs using the MDeePred method (Table S4). After reviewing the current literature on these 380 drug–target interactions, six were chosen for further investigation (Table 2). Among these, five proteins associated with HCC carcinogenesis were identified: FGFR1, ALK, AKT1, FLT3, and PI3K. Each of these target proteins plays crucial roles in various metabolic processes, and their dysfunctions contribute to the development and progression of HCC. Fibroblast growth factor receptor 1 (FGFR1) belongs to the type 4 receptor tyrosine kinase family (FGFR1–4), which binds to fibroblast growth factors (FGFs) [45]. Overexpression of FGFR1 has been found to have important roles in HCC [46,47]. Anaplastic lymphoma kinase (ALK) is a significant molecular target in the receptor tyrosine kinase family, holding vast relevance in drug discovery, particularly for cancer treatments. ALK is a member of the insulin receptor superfamily and plays a role in multiple malignancies, HCC being one of them [48,49]. The PI3K/Akt/mTOR signaling pathway promotes cell growth, invasion, and angiogenesis and prevents cell apoptosis in various cancers [48]. Loss of the PTEN tumor suppressor protein leads to hyperactivity in the PI3K/Akt pathway, which promotes cell survival and resistance to therapeutics in various cancers, including liver cancer [50,51]. FLT3 is a receptor tyrosine kinase, and its inhibition has been shown to reduce tumor size in HCC, making it a promising therapeutic target for treatment [52,53].

The enrichment target proteins that are selected as a result of MDeePred prediction resulted in molecular function classification into two main categories: transmembrane receptor protein tyrosine kinase activity (Figure 1) and ATP binding (Figure 2). Meanwhile, analyses of biological processes were categorized into 27 primary groups (Figure 3). To the best of our knowledge, the predicted drug molecules have never been tested on these target proteins with respect to HCC. After that, SwissADME (Figure 4 and Figure 5 and Table 3 and Tables S5–S8) and the molecular docking properties (Figure 6 and Table 4) were determined for six DTIs that contain five different targets, six drug candidates; and HCC-approved drugs (lenvatinib, regorafenib, and sorafenib).

The oral bioavailability radar offers a brief evaluation of a compound’s drug-likeness by evaluating six physicochemical properties: saturation, lipophilicity, polarity, size, solubility, and flexibility [54]. The lipophilicity (XLOGP3) ranged between −0.7 and +5.0, and molecular weights were between 150 and 500. Polarity, defined by TPSA, ranged from 20 to 130 Å^2^, while solubility (log S) did not exceed 6. The saturation, indicated by the fraction of carbons in sp^3^ hybridization, was not less than 0.25, and flexibility was defined by a maximum of nine rotatable bonds (Figure 4 and Table S5) [55]. CHEMBL388978, CHEMBL328029, and CHEMBL1165499 fall within the favorable zone for lipophilicity, size, polarity, solubility, saturation, and flexibility. CHEMBL1615189 and CHEMBL1773601 meet all the criteria, except for saturation. CHEMBL1773581 meets all the criteria, except for saturation and polarity. The analyzed drugs approved for HCC treatment, lenvatinib, regorafenib, and sorafenib, meet all the criteria, except saturation. 

In the BOILED-Egg diagram (Figure 5), the selected compounds within the white ellipse indicate potential for GIA. Those in the yellow ellipse, or “yolk”, suggest a strong likelihood of crossing the BBB to access the central nervous system (CNS) [56]. CHEMBL388978, CHEMBL328029, and CHEMBL1165499, as shown in the diagram, demonstrate high BBB penetration and GIA. Only lenvatinib demonstrated high GIA.

The methods iLOG, XLOGP3, WLOGP, MLOGP, and SILICOS-IT were used to estimate the Log Po/w values for the compounds. These different methods represent various methodologies to estimate how lipophilic (or hydrophobic) a compound is [57,58,59,60,61,62]. The consensus Log Po/w value is calculated as the arithmetic mean of the predictions made by these five methods. This average provides a more reliable estimate by balancing out the potential biases or errors of individual methods [57]. The Log Po/w is ranged between −0.7 and +5.0 according to the oral bioavailability radar. This range is significant for determining the oral bioavailability of these compounds. The specific Log Po/w values for CHEMBL388978, CHEMBL1615189, CHEMBL328029, CHEMBL1165499, CHEMBL1773581, and CHEMBL1773601 are reported in Table S6, all falling within the acceptable range, indicating favorable characteristics for oral absorption. Lenvatinib falls within the acceptable range. Regorafenib and sorafenib fall within the acceptable range, except for Log Po/w (WLOGP). The water solubility of the compounds is categorized using Log S values, which range from insoluble (−10) to highly soluble (0) [63,64]. The Log S values of CHEMBL388978, CHEMBL1615189, CHEMBL328029, CHEMBL1165499, CHEMBL1773581, CHEMBL1773601, lenvatinib, regorafenib, and sorafenib are given in Table S7. CHEMBL388978, CHEMBL1165499, and CHEMBL1773581 are within the acceptable range for the moderately soluble class. CHEMBL328029 is within the acceptable range for the soluble class. CHEMBL1615189 and CHEMBL1773601 are within the acceptable range for the poorly soluble class. Moreover, lenvatinib, regorafenib, and sorafenib are within the acceptable range for the moderately soluble class.

The drug-likeness of the candidate compounds is evaluated using SwissADME, which applies rule-based filters and the Abbot bioavailability score to determine their suitability based on key pharmacokinetics criteria (Table 3) [65]. CHEMBL388978, CHEMBL1615189, and CHEMBL1773581 meet several of these criteria, with each compound adhering to different combinations of the Lipinski, Veber, Egan, and Muegge rules. CHEMBL328029 and CHEMBL1165499 show broader compliance, aligning with nearly all filters, except Ghose in the case of CHEMBL1165499. Meanwhile, CHEMBL1773601 satisfies all but the Egan rule. Lenvatinib shows broader compliance, aligning with all filters. Regorafenib and sorafenib satisfy all but the Ghose and Egan rules. These assessments indicate that the compounds possess characteristics favorable for drug development, with varying degrees of alignment to the established pharmacokinetic rules.

Pan-assay interference compounds (PAINS) are known for their problematic nonspecific interactions with multiple biological targets, which can lead to misleading outcomes in drug discovery [66]. Another tool used in the assessment is the Brenk filter, which helps identify unwanted functionalities that might contribute to potential toxicity or unfavorable pharmacokinetics. CHEMBL388978, CHEMBL1165499, CHEMBL1773581, CHEMBL1773601, lenvatinib, regorafenib, and sorafenib passed this filter without any alerts, while CHEMBL1615189 and CHEMBL328029 each had one alert, suggesting some concerns regarding their functional groups, as noted in Table S8. Lead-likeness is another important criterion, focusing on the overall suitability of a compound as a starting point for drug development. Here, only CHEMBL328029 met all the criteria with no violations, indicating its potential as a promising lead candidate. Furthermore, the synthetic accessibility of these compounds, which measures how easily they can be produced using standard synthetic methods, indicates that all are relatively easy to synthesize [57]. CHEMBL328029 stands out as particularly accessible, making it not only a lead-like but also a synthetically feasible candidate for further development. This combination of favorable properties makes CHEMBL328029 a standout in the group, despite the single alert in the Brenk filter.

Molecular docking is an important tool used to predict the binding behaviors of small molecules to their target proteins, identifying potential sites and affinities crucial for drug development [67,68]. Here, the docking results provide insights into the molecular interactions specific to HCC for the MDeePred DTIs. The visual molecular interactions in Figure 6 and vina scores in Table 4 indicate that the compounds have favorable docking properties for their protein targets. The greater the negative value of the vina score, the greater the Gibbs binding energy for drug–target complexes. This increases the binding potential of drug–target complexes. Contact residues and bonds showed contact amino acids and bond structures between the ligands and target proteins. The vina scores determined for lenvatinib, regorafenib, and sorafenib used in the treatment of advanced HCC and the vina scores of the six small molecules highlighted in our study were compared. The negative vina score (or Gibbs binding energy) of CHEMBL1165499 was found to be higher than sorafenib and equal to regorafenib for ALK. The negative vina scores of CHEMBL1773601 and CHEMB1773581 were found to be higher than all the drugs for AKT1. The negative vina score of CHEMBL388978 was found to be higher than lenvatinib and regorafenib for FLT3. The negative vina score of CHEMBL1615189 was found to be higher than lenvatinib for PIK3CA. As a result of the comparisons made with lenvatinib, regorafenib, and sorafenib used in the treatment of advanced HCC, it was determined that the six small molecules featured in our study are promising drug candidates to be used in the treatment of HCC.

Finally, we performed a literature survey on the MDeePred predicted small molecules (Table 5) [37,38,39,40]. The pairing of FGFR1 with CHEMBL328029 was reported as the potential biological target of small molecules using in silico repositioning strategies, ligand-based similarity predictions, and molecular docking analyses. Additionally, the ALK and its potential binding molecule, CHEMBL1165499, were described as new kinases for therapeutic drug targets. Molecules CHEMBL1773601, CHEMBL1773581, and CHEMBL1615189 targeting AKT1 and PIK3CA were reported as a result of the structure of selective kinase inhibitors using molecular modeling and 3D-QSAR methods. Validated with experimental data, the model demonstrated high reliability in predicting the effectiveness of these inhibitors. Staurosporine, a well-known multi-kinase inhibitor effective at micromolar concentrations, shows target specificity at lower concentrations. The interaction between CHEMBL388978 (staurosporine) and FLT3 was reported to have bioactivity at nanomolar concentrations. 

The current treatments for HCC vary depending on the disease stage, tumor size, the patient’s overall health, and liver function. However, the widely accepted treatments include surgical resection, liver transplantation, local ablative therapies, TACE, molecular targeted therapies, and immunotherapy. Our study focuses on molecular targeted therapies, specifically tyrosine kinase inhibitors like sorafenib and lenvatinib, which are drug treatments approved for advanced HCC. These small molecule agents work by inhibiting tumor growth and angiogenesis [69,70,71,72,73]. Our study identified six small molecules, all interacting with genes exhibiting kinase activity, including FGFR1, ALK, and FLT3 proteins with tyrosine kinase activity. These findings suggest that the six small molecules could be further exploited as kinase or tyrosine kinase inhibitors in the treatment of HCC. These potential drug candidates will be available for clinical use following in vitro and in vivo studies.

In this study, small molecule drug candidates for HCC treatment were identified using the machine learning-based in silico MDeePred method for drug repurposing. We demonstrated that machine learning tools can be effectively used for drug repurposing in HCC to identify potential new therapeutic agents that carry highly drug-like properties similar to those of HCC-approved drugs. Consequently, the MDeePred-based drug repurposing method provided new drug candidates for HCC that can be less costly and time-consuming. Our approach using the MDeePred method can also be applied to other types of cancer.

## 4. Materials and Methods

### 4.1. Data Collection

HCC-related genes were identified using the Open Targets Platform database. The Open Targets Platform is used as a powerful tool to find disease-associated genes. By integrating a wide range of information from genetic data, clinical findings, and biomedical databases, it helps to identify potential genetic targets contributing to the etiology of a disease [74]. Genetic associations and somatic mutations were chosen as data-type filters. The arithmetic mean of the genetic associations and somatic mutations was calculated to select the actual HCC-related genes to be used in this research [75]. The gene list is given in Table S1.

The UniProt database was used to verify the protein products of the selected HCC-related genes. UniProt is a protein database that provides comprehensive, high-quality information on protein sequences and functions for biological research [76]. The ChEMBL database was used to construct the manually curated compound–protein activity dataset for each HCC-related gene (called targets during this study). ChEMBL is a chemical database that provides comprehensive information on small molecules with known biological activities and their potential in drug discovery [77]. First, datasets were filtered with respect to the “target organism” (i.e., *Homo sapiens*), “target type” (i.e., single protein), “assay type” (i.e., binding assays), “standard unit” (i.e., molar), “standard type” [i.e., the half maximal inhibitory concentration (IC_50_)], and “standard relation” (i.e., = and >) attributes (Table S3). We noticed that the dataset contained repeated measurements from separate experiments. To handle this, we calculated the median bioactivity for each pair and used this as the single bioactivity measurement. We then excluded the bioactivity measurements without pChEMBL value, which represents the half-maximal response on a negative logarithmic scale. A data point with a pChEMBL value indicates that the corresponding record has been curated and is thus considered reliable [78]. Following data filtration (filtered gene sets are given in Table S3), the dataset was grouped using the UniProt, ChEMBL, and Expasy databases. We selected the “HCC-associated transferases” enzyme class as our final dataset to employ MDeePred drug target deep learning-based binding affinity prediction in the tool [12,79,80]. 

The rationale behind the selection of transferases lies in their critical involvement in transferring functional groups, such as phosphate, methyl, or hydroxyl groups. Transferases play pivotal roles in modulating protein function and activity, which are indispensable processes in the context of carcinogenesis. Phosphate transferases, for instance, regulate signal transduction pathways by phosphorylating proteins, thereby influencing cell growth and differentiation. Similarly, methyl and hydroxyl transferases contribute to epigenetic modifications and post-translational modifications of proteins, ultimately impacting gene expression and cellular functions associated with cancer development and progression. By targeting transferases involved in these essential molecular mechanisms, we aim to gain insights into their potential as therapeutic targets and elucidate their roles in driving oncogenic processes.

### 4.2. Data Preperation for MDeePred and Selection of the DTIs

The MDeePred method was employed as a deep learning tool to identify the eventual drug candidates for HCC. For the MDeePred method, training and test datasets were formed using the “transferases” bioactivity drug target data, according to Rifaioglu et al. [12]. In MDeePred, each compound is depicted as a 2D image of 200 × 200 pixels, showing its molecular structure from their SMILES strings. SMILES is a standardized representation available in open-access bioactivity data repositories, which includes all the necessary information for generating the 2D images. 

We then used the MDeePred tool trained specifically for the “HCC-associated transferases” dataset to screen over a million small molecule drug compound entries from the ChEMBL database (v24) to predict novel DTIs. Subsequent to this, a statistical measure was undertaken to assign the bioactivities of small molecules of the targets within the extensive DTI predictions. We conducted an ontology-based enrichment test, specifically for protein sets, to discern the shared characteristics of these targets. In this analysis, annotations were overrepresented based on GO molecular function and biological process ontology terms were prioritized based on their statistical relevance on target proteins [81]. 

### 4.3. In Silico Validation of Predicted Small Molecule Target HCC Transferases

Using SwissADME online, the small molecule drug candidate compounds against “HCC-associated transferases” were analyzed for their physicochemical attributes, lipophilicity, water solubility, drug-likeness, and medicinal chemistry tool [57]. Molecular docking was conducted using CB-Dock version 2, a web server. Blind docking was executed by inputting the 3D structure PDB file of five distinct targets alongside the SDF file of each drug compound into the server. The analysis was focused on the docking poses that had the highest vina scores [82]. In addition, comparisons were made with our small molecules for lenvatinib, regorafenib, and sorafenib, which are currently used in the treatment of advanced HCC.

### 4.4. Literature-Based Validation of Novel DTI Predictions towards Drug Repurposing

We carried out a literature search to validate the predictions “HCC-associated transferases” DTI pairs from MDeePred to support the evidence of DTIs in published scientific reports. We focused on the interactions between target proteins and drugs in order to prove the experimental validation of our drug repurposing DTIs.

## Figures and Tables

**Table 1:** Identification of 22 targets as transferases.

Genes	ChEMBL ID	Genes Name
*ACVR2A*	CHEMBL5616	Activin receptor type-2A
*AKT1*	CHEMBL4282	Serine/threonine-protein kinase AKT
*ALK*	CHEMBL4247	ALK tyrosine kinase receptor
*ATM*	CHEMBL3797	Serine-protein kinase ATM
*CREBBP*	CHEMBL5747	CREB-binding protein
*ERBB3*	CHEMBL5838	Receptor tyrosine-protein kinase erbB-3
*FGFR1*	CHEMBL3650	Fibroblast growth factor receptor 1
*FLT3*	CHEMBL1974	Tyrosine-protein kinase receptor FLT3
*FLT4*	CHEMBL1955	Vascular endothelial growth factor receptor 3
*JAK3*	CHEMBL2148	Tyrosine-protein kinase JAK3
*KDR*	CHEMBL279	Vascular endothelial growth factor receptor 2
*KIT*	CHEMBL1936	Stem cell growth factor receptor
*KMT2A*	CHEMBL1293299	Histone-lysine N-methyltransferase MLL
*MAP3K1*	CHEMBL3956	Mitogen-activated protein kinase kinase kinase 1
*MET*	CHEMBL3717	Hepatocyte growth factor receptor
*NTRK1*	CHEMBL2815	Nerve growth factor receptor Trk-A
*PIK3CA*	CHEMBL4005	PI3-kinase p110-alpha subunit
*PRKACA*	CHEMBL4101	cAMP-dependent protein kinase alpha-catalytic subunit
*RET*	CHEMBL2041	Tyrosine-protein kinase receptor RET
*ROS1*	CHEMBL5568	Proto-oncogene tyrosine-protein kinase ROS
*SETD2*	CHEMBL3108647	Histone-lysine N-methyltransferase SETD2
*TERT*	CHEMBL2916	Telomerase reverse transcriptase

**Table 2:** Selected drug–target interactions list.

Ligand (Drug/Compound)	Molecular Formula of Ligands	2D Structure of Ligands	Target Protein
CHEMBL388978 (Staurosporine)	C28H26N4O3	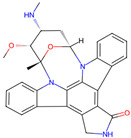	Tyrosine-protein kinase receptor FLT3 (FLT3)
CHEMBL1615189	C20H14ClFN4O3S2	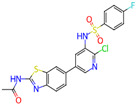	PI3-kinase p110-alpha subunit (PIK3CA)
CHEMBL328029	C17H16N2O	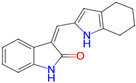	Fibroblast growth factor receptor 1 (FGFR1)
CHEMBL1165499	C24H26F2N6	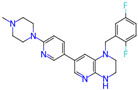	ALK tyrosine kinase receptor (ALK)
CHEMBL1773581	C20H17N5O3S2	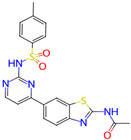	Serine/threonine-protein kinase AKT (AKT1)
CHEMBL1773601	C22H19N3O4S2	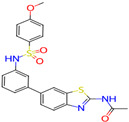	Serine/threonine-protein kinase AKT (AKT1)

**Table 3:** Drug-likeness of the drug candidate compounds and drugs (lenvatinib, regorafenib, and sorafenib).

Drug Candidate Compounds	Lipinski	Ghose	Veber	Egan	Muegge	Bioavailability Score
CHEMBL388978	Yes; 0 violation	No; 1 violation: MR > 130	Yes	Yes	No; 1 violation: #rings > 7	0.55
CHEMBL1615189	Yes; 0 violation	No; 1 violation: WLOGP > 5.6	Yes	No; 2 violations: WLOGP > 5.88, TPSA > 131.6	Yes	0.55
CHEMBL328029	Yes; 0 violation	Yes	Yes	Yes	Yes	0.55
CHEMBL1165499	Yes; 0 violation	No; 1 violation: MR > 130	Yes	Yes	Yes	0.55
CHEMBL1773581	Yes; 0 violation	Yes	No; 1 violation: TPSA > 140	No; 1 violation: TPSA > 131.6	No; 1 violation: TPSA > 150	0.55
CHEMBL1773601	Yes; 0 violation	Yes	Yes	No; 1 violation: TPSA > 131.6	Yes	0.55
Lenvatinib	Yes; 0 violation	Yes	Yes	Yes	Yes	0.55
Regorafenib	Yes; 0 violation	No; 2 violations: MW > 480, WLOGP > 5.6	Yes	No; 1 violation: WLOGP > 5.88	Yes	0.55
Sorafenib	Yes; 0 violation	No; 1 violation: WLOGP > 5.6	Yes	No; 1 violation: WLOGP > 5.88	Yes	0.55

**Table 4:** Docking results of the ligands; MDeePred DTI transferases; and approved drugs: lenvatinib, regorafenib, and sorafenib.

Ligand (Drug/Compound)	Target Protein	Vina Score	Cavity Volume (Å^3^)	Contact Residues
CHEMBL328029	FGFR1	−8.7	543	ILE19 GLN24 LYS51 LEU54 PHE55 GLY58 GLN59 ILE61 MET62 VAL75 PHE91 VAL93 HIS96 ILE99 TYR100
CHEMBL1165499	ALK	−9.6	6263	VAL131 ASP133 GLU135 VAL136 ASN426 ILE427 ASN428 MET441 ALA442 LEU443 TRP446 VAL461 THR462 GLY463 SER464 LYS468 LEU639 LYS640 GLU642 GLN643 LEU645 THR679 VAL680 SER681 GLN682 ARG683
CHEMBL1773601	AKT1	−7.8	110	LYS8 GLU9 GLY10 TRP11 LEU12 HIS13 PRO24 TYR26 ARG41 VAL90 GLU91 GLU95 TRP99
CHEMBL1773581	AKT1	−7.4	110	LYS8 GLU9 GLY10 TRP11 LEU12 HIS13 PRO24 HIS89 VAL90 GLU91 GLU95 TRP99
CHEMBL388978	FLT3	−9.6	1932	GLY1121 LEU1122 GLY1123 HIS1124 GLY1125 VAL1130 ALA1148 LYS1150 VAL1180 LEU1196 GLU1197 LEU1198 MET1199 GLY1202 ASP1203 ARG1253 ASN1254 LEU1256 GLY1269 ASP1270
CHEMBL1615189	PIK3CA	−9.6	904	LEU484 GLY485 VAL492 ALA512 LYS514 GLU531 MET535 ILE545 VAL561 GLU562 TYR563 ALA564 GLY567 LEU630 ALA640 ASP641
Lenvatinib	FGRF1	−9.1	8731	ARG576 ARG577 PRO578 LEU595 SER596 SER597 LEU600 TRP691 PHE694 THR695 LEU696 GLY698 SER699 TYR701 PRO702 HIS717 ARG718 MET719 ASP720 LYS721 PRO722 SER723 ASN724 TYR730 ARG577 PRO578 LEU595 LEU600 TRP691 PHE694 THR695 LEU696 GLY697 GLY698 SER699 PRO700 TYR701 PRO702 HIS717 ARG718 MET719 ASP720 LYS721 PRO722 SER723 ASN724 ASN727
Lenvatinib	ALK	−9.0	1932	ARG1120 LEU1122 GLY1123 HIS1124 GLY1125 ALA1126 VAL1130 GLU1132 ALA1148 VAL1149 LYS1150 VAL1180 LEU1196 GLU1197 LEU1198 MET1199 ALA1200 GLY1201 GLY1202 ASP1203 LYS1205 SER1206 ASP1249 ARG1253 ASN1254 CYS1255 LEU1256 GLY1269 ASP1270 GLY1272 MET1290
Lenvatinib	AKT1	−6.7	110	VAL7 LYS8 GLU9 GLY10 TRP11 LEU12 HIS13 PRO24 ARG25 TYR26 ARG41 HIS89 VAL90 GLU91 GLU94 GLU95 GLU98 TRP99
Lenvatinib	FLT3	−8.5	832	TYR572 GLU573 SER574 GLN575 TYR589 TYR591 PHE621 ALA657 ARG810 ASP811 ASN816 ASP829 PHE830 GLY831 LEU832 ARG834 ILE836 TYR842 ARG845 GLY846 ASN847 ALA848 ARG849 LEU850 PRO851 MET855 SER859 LEU860 PHE861 GLU862 GLY863 ILE864 TYR865
Lenvatinib	PIK3CA	−8.9	6263	GLU127 MET130 VAL131 LYS132 ASP133 PRO134 GLU135 VAL136 ASN426 ILE427 ASN428 PHE430 ASP431 TYR432 THR435 LEU436 VAL437 SER438 MET441 ALA442 LEU443 TRP446 VAL461 THR462 GLY463 SER464 ASN465 PRO466 LYS468 LYS640 GLU642 GLN643 TYR644 LEU645 THR679 VAL680 GLN682 ARG683
Regorafenib	FGRF1	−9.9	8731	GLN574 ARG577 PRO578 TRP691 PHE694 THR695 LEU696 GLY698 SER699 PRO700 TYR701 PRO702 VAL704 HIS717 ARG718 MET719 ASP720 LYS721 PRO722 SER723 ASN724 TYR730 ARG577 PRO578 LEU595 SER597 LEU600 TRP691 PHE694 THR695 LEU696 GLY697 GLY698 SER699 PRO700 TYR701 PRO702 VAL704 LEU712 GLU715 GLY716 HIS717 ARG718 MET719 ASP720 LYS721 PRO722 SER723 ASN724 ARG734
Regorafenib	ALK	−9.6	1932	ARG1120 GLY1121 LEU1122 GLY1123 HIS1124 GLY1125 ALA1126 VAL1130 GLU1132 GLN1146 ALA1148 LYS1150 VAL1180 LEU1196 GLU1197 LEU1198 MET1199 ALA1200 GLY1201 GLY1202 ASP1203 LYS1205 SER1206 ASP1249 ARG1253 ASN1254 CYS1255 LEU1256 GLY1269 ASP1270 GLY1272 MET1273 MET1290
Regorafenib	AKT1	−7.2	110	ILE6 VAL7 LYS8 GLU9 GLY10 TRP11 LEU12 HIS13 PRO24 ARG25 TYR26 LYS39 GLU40 ARG41 HIS89 VAL90 GLU91 GLU95 GLU98 TRP99 THR101 ALA102 THR105
Regorafenib	FLT3	−8.6	832	TYR572 GLU573 SER574 GLN575 LEU576 GLN577 MET578 TYR589 TYR591 VAL592 ASP593 PHE594 ARG595 PHE621 LEU646 ARG655 GLU656 ALA657 SER660 GLU661 MET664 ARG810 ASP811 ASN816 ASP829 PHE830 GLY831 LEU832 ARG834 ILE836 TYR842 ASN847 ALA848 ARG849 LEU850 PRO851 MET855 SER859 LEU860 GLU862 GLY863 TYR865
Regorafenib	PIK3CA	−10.0	2313	TYR165 VAL166 TYR167 PRO168 ASN170 VAL196 ILE197 TYR250 LYS253 VAL254 CYS257 ASP258 GLU259 TYR260 LYS271 TYR272 SER275 MET286 LEU287 MET288 ALA289 SER292 SER295 GLN296 LEU297 PRO298 GLN661 ARG662 HIS665 PHE666 MET697 TYR698 HIS701 GLY750 PHE751 LEU752 ASN756 PRO757 ALA758 HIS759 GLN760 LEU761 GLY762 PRO786 ASP787 ILE788 LEU793 PHE794
Sorafenib	FGRF1	−9.9	348	LEU484 GLY485 GLU486 GLY487 ALA488 PHE489 GLY490 GLN491 VAL492 ALA512 VAL513 LYS514 MET515 LEU516 ASP524 ASP527 LEU528 GLU531 MET535 ILE545 VAL559 VAL561 GLU562 TYR563 ALA564 GLY567 ASN568 ARG570 GLU571 ARG627 ASN628 LEU630 ILE639 ALA640 ASP641 PHE642 LEU644 ALA645 THR657 THR658 ASN659
Sorafenib	ALK	−8.9	1932	ARG1120 LEU1122 GLY1123 HIS1124 GLY1125 ALA1126 VAL1130 GLU1132 ALA1148 VAL1149 LYS1150 VAL1180 LEU1196 GLU1197 LEU1198 MET1199 ALA1200 GLY1202 ASP1203 LYS1205 SER1206 GLU1210 ASP1249 ARG1253 ASN1254 LEU1256 GLY1269 ASP1270 GLY1272 MET1273
Sorafenib	AKT1	−7.0	152	LYS14 ARG15 GLY16 GLU17 TYR18 ILE19 LYS20 ARG23 LEU52 ASN53 ASN54 PHE55 THR65 GLU66 ARG67 PRO68 THR72 ILE74 ARG76 GLN79 THR82 VAL83 ILE84 GLU85 ARG86 THR87
Sorafenib	FLT3	−10.3	832	TYR572 GLU573 SER574 GLN575 LEU576 GLN577 MET578 TYR591 VAL592 ASP593 PHE594 ARG595 PHE621 GLU656 ALA657 SER660 GLU661 MET664 ARG810 ASP811 ASN816 ASP829 PHE830 GLY831 LEU832 ARG834 ILE836 TYR842 ARG845 GLY846 ASN847 ALA848 ARG849 LEU850 PRO851 MET855 SER859 LEU860 GLU862 GLY863 ILE864 TYR865
Sorafenib	PIK3CA	−10.3	2313	TYR165 VAL166 TYR167 PRO168 PRO169 ASN170 ASP258 GLU259 TYR260 MET288 SER292 LEU293 GLN296 LEU297 PRO298 ASP300 GLN661 ARG662 HIS665 CYS695 GLY696 MET697 TYR698 LYS700 HIS701 GLY750 PHE751 LEU752 ASN756 PRO757 ALA758 GLN760

**Table 5:** Literature verified the selected DTI predictions of MDeePred.

Ligand (Drug/ Compound)	Target Protein	Experimental Bioactivity	Reference
CHEMBL328029	Fibroblast growth factor receptor 1 (FGFR1)	IC_50_: 10,500 nM	[37]
CHEMBL1165499	ALK tyrosine kinase receptor (ALK)	IC_50_: 33 nM	[38]
CHEMBL1773601	Serine/threonine-protein kinase AKT (AKT1)	IC_50_: 1160 nM	[39]
CHEMBL1773581	Serine/threonine-protein kinase AKT (AKT1)	IC_50_: 1260 nM	[39]
CHEMBL388978 (Staurosporine)	Tyrosine-protein kinase receptor FLT3 (FLT3)	IC_50_: 1 nM	[40]
CHEMBL1615189	PI3-kinase p110-alpha subunit (PIK3CA)	IC_50_: 6.3 nM	[39]

## Data Availability

The original contributions presented in the study are included in the article/Supplementary Materials. Further inquiries can be directed to the corresponding author.

## Supplementary Materials

The following supporting information can be downloaded at https://www.mdpi.com/article/10.3390/ijms25179392/s1.
