# Supplementary material for: Drug Repurposing Approach to Identify Candidate Drug Molecules for Hepatocellular Carcinoma
Source: Int J Mol Sci. 2024 Aug 29;25(17):9392. doi: 10.3390/ijms25179392 (PMC11395636; doi:10.3390/ijms25179392)
Supplement: Supplementary file 1 [file ijms-25-09392-s001.zip › ijms-3169798-supplementary.pdf]

Supplementary Table S1. All targets associated with HCC after data type filtering.

Target Symbol	Target Name	Overall Association Scores	Genetic Association Scores	Somatic Mutation Scores	Drug Scores	Pathways & Systems Biology Scores	RNA Expression Scores	Text Mining Scores	Animal Model Scores
TP53	tumor protein p53	1.0	1.0	1.0	0.0	0.7152195485738936	0.0	0.30033021847080205	0.25
CTNNB1	catenin beta 1	1.0	1.0	1.0	0.0	0.6581859646946654	0.0	0.23098058920399783	0.0
AXIN1	axin 1	1.0	0.0	1.0	0.0	1.0	0.0	0.12303580566311544	0.0
MET	MET proto-oncogene, receptor tyrosine kinase	1.0	0.0	0.9915123456790124	1.0	0.0	0.0	0.04230123609121459	0.0
FGFR1	fibroblast growth factor receptor 1	1.0	0.0	0.798611111111111	1.0	0.0	0.0	0.21770072794453407	0.0
KDR	kinase insert domain receptor	1.0	0.0	0.5059156378600823	1.0	0.25	0.0	0.20383866416785226	0.0
PDGFRA	platelet derived growth factor receptor alpha	1.0	0.0	0.48055555555555557	0.9993859229924646	0.0	0.10823473706565383	0.2626506472001884	0.0
RAF1	Raf-1 proto-oncogene, serine/threonine kinase	1.0	0.0	0.4814814814814815	1.0	0.25	0.0	0.105406028785698	0.0
FLT3	fms related tyrosine kinase 3	1.0	0.0	0.5505952380952381	1.0	0.0	0.0	0.04895905328798186	0.0
PDGFRB	platelet derived growth factor receptor beta	1.0	0.0	0.48194444444444445	1.0	0.0	0.0	0.21368191472270465	0.0
FLT4	fms related tyrosine kinase 4	1.0	0.0	0.5326829805996472	1.0	0.0	0.0	0.09394877194955117	0.0
RET	ret proto-oncogene	1.0	0.0	0.5042438271604939	1.0	0.0	0.02796321498196705	0.11195600901584819	0.0
FGFR2	fibroblast growth factor receptor 2	1.0	0.0	0.4166666666666667	1.0	0.0	0.10296000000000001	0.2279852117105633	0.0
KIT	KIT proto-oncogene, receptor tyrosine kinase	1.0	0.0	0.5326829805996472	1.0	0.0	0.0	0.006	0.0
BRAF	B-Raf proto-oncogene, serine/threonine kinase	1.0	0.0	0.4166666666666667	1.0	0.0	0.009590970835463432	0.14071122072090142	0.0
PIK3CA	phosphatidylinositol-4,5-bisphosphate 3-kinase catalytic subunit alpha	1.0	0.5	1.0	0.1	0.25	0.0	0.14530945936311657	0.0
CD274	CD274 molecule	1.0	0.0	0.2777777777777778	1.0	0.0	0.0	0.31453379905487555	0.0
FGFR4	fibroblast growth factor receptor 4	1.0	0.0	0.2777777777777778	1.0	0.0	0.0	0.2819590336600308	0.0
FGFR3	fibroblast growth factor receptor 3	1.0	0.0	0.2777777777777778	1.0	0.0	0.0	0.10701768123254814	0.0
TERT	telomerase reverse transcriptase	1.0	0.8419444444444443	0.711398898898899	0.0	0.0	0.06114595711989072	0.2998225453076396	0.0
TOP1	DNA topoisomerase I	1.0	0.0	0.1388888888888889	1.0	0.0	0.0	0.155899933106576	0.0
EGFR	epidermal growth factor receptor	0.988903369037091	0.0	0.48042929292929293	0.8182777351054156	0.25	0.015633249479851088	0.30841182648142323	0.0
NFE2L2	nuclear factor, erythroid 2 like 2	0.9861488324613179	0.0	0.9710778486820153	0.0	0.0	0.0	0.07905584448256438	0.19054000000000001
IDH1	isocitrate dehydrogenase (NADP(+)) 1, cytosolic	0.9757839263791365	0.0	0.9635802469135802	0.0	0.0	0.016559116501942018	0.18466103688765695	0.0
HRAS	HRas proto-oncogene, GTPase	0.9655228070697964	0.0	0.947787628425856	0.0	0.0	0.0	0.15961660779546386	0.0
CDKN2A	cyclin dependent kinase inhibitor 2A	0.9643727643583659	0.0	0.9375694656423823	0.0	0.0	0.10561075816007524	0.1818236369788107	0.0
ESR1	estrogen receptor 1	0.9519781878015823	0.0	0.503343621399177	0.75	0.5	0.11194458618510383	0.2577430951812522	0.0
POLE	DNA polymerase epsilon, catalytic subunit	0.9355827938466809	0.0	0.48023504273504275	0.8155240331629202	0.0	0.0	0.0	0.0
CREBBP	CREB binding protein	0.9353246276167473	0.0	0.9284357387278583	0.0	0.0	0.0	0.062	0.0
IDH2	isocitrate dehydrogenase (NADP(+)) 2, mitochondrial	0.9276793075494287	0.0	0.911033950617284	0.0	0.0	0.0	0.14980821238930225	0.0

NRAS	NRAS proto-oncogene, GTPase	0.9276289828184364	0.0	0.917283950617284	0.0	0.0	0.00809438597841138	0.08855219769751527	0.0
POLD1	DNA polymerase delta 1, catalytic subunit	0.9254042800765004	0.0	0.4166666666666667	0.8155240331629202	0.0	0.0	0.05142222222222223	0.0
ARID2	AT-rich interaction domain 2	0.8975792476262491	0.0	0.8680265222849968	0.0	0.0	0.0	0.2659745280712706	0.0
CSF1R	colony stimulating factor 1 receptor	0.8958985846956575	0.0	0.3101851851851852	0.812300883239736	0.0	0.005214491736277451	0.05152949483497103	0.0
GNAS	GNAS complex locus	0.8643253914380926	0.0	0.8603395061728395	0.0	0.0	0.007063053132937941	0.031900000000000005	0.0
APC	APC regulator of WNT signaling pathway	0.8617515044760831	0.0	0.744624732905983	0.0	0.6341215558174885	0.0	0.0	0.0
DDR2	discoidin domain receptor tyrosine kinase 2	0.8548531243600074	0.0	0.4166666666666667	0.725	0.0	0.015348453744889225	0.22254461400856643	0.0
ABL1	ABL proto-oncogene 1, non-receptor tyrosine kinase	0.8530055555555555	0.0	0.2777777777777778	0.767361111111111	0.0	0.0	0.14579999999999999	0.0
KRAS	KRAS proto-oncogene, GTPase	0.8270290899955751	0.0	0.8159722222222222	0.0	0.0	0.0	0.09951180996017617	0.0
TSC2	TSC complex subunit 2	0.8223728912477103	0.0	0.7777111760883691	0.0	0.0	0.0	0.17864686063736468	0.0
SF3B1	splicing factor 3b subunit 1	0.7874111111111111	0.0	0.7638888888888888	0.0	0.0	0.0	0.2117	0.0
IL6ST	interleukin 6 signal transducer	0.7595346974403909	0.0	0.7105475040257648	0.0	0.0	0.0	0.1959487736585046	0.0
ARID1A	AT-rich interaction domain 1A	0.7282025652730801	0.0	0.702991252261052	0.0	0.0	0.0	0.22690181710825336	0.0
HLA-DQA1	major histocompatibility complex, class II, DQ alpha 1	0.7225721750469736	0.7225721750469736	0.0	0.0	0.0	0.0	0.0	0.0
IGF2R	insulin like growth factor 2 receptor	0.7190700746893237	0.0	0.6944444444444444	0.0	0.0	0.0	0.09850252097951709	0.0
PTEN	phosphatase and tensin homolog	0.7142386544095812	0.0	0.661028972520908	0.0	0.29049471895720985	0.0	0.315483857584629	0.0
RXRA	retinoid X receptor alpha	0.7071105414537384	0.0	0.6944444444444444	0.0	0.0	0.0	0.05066438803717578	0.0
TCF7L2	transcription factor 7 like 2	0.7060830726587126	0.0	0.530542695473251	0.0	0.5604923708790421	0.0	0.11659525120221949	0.0
KIF1B	kinesin family member 1B	0.6995011950776868	0.651450028120844	0.0	0.0	0.0	0.0	0.1922046678273713	0.0
CNOT9	CCR4-NOT transcription complex subunit 9	0.6957521220793593	0.0	0.6944444444444444	0.0	0.0	0.005230710539659667	0.0	0.0
CASP8	caspase 8	0.6951975105906835	0.0	0.6756365740740741	0.0	0.0	0.0	0.17604842864948486	0.0
SF3B2	splicing factor 3b subunit 2	0.6793944444444445	0.0	0.6770833333333334	0.0	0.0	0.0	0.020800000000000003	0.0
NF1	neurofibromin 1	0.6730377888636936	0.0	0.6680555555555555	0.0	0.0	0.0	0.04484009977324263	0.0
ACVR2A	activin A receptor type 2A	0.6696192495126705	0.0	0.6684636939571149	0.0	0.0	0.0	0.010400000000000001	0.0
KMT2C	lysine methyltransferase 2C	0.6657109658678286	0.0	0.6634331880900508	0.0	0.0	0.0	0.0205	0.0
PBRM1	polybromo 1	0.6632685273368606	0.0	0.6490575396825397	0.0	0.0	0.0	0.12789888888888887	0.0
ATM	ATM serine/threonine kinase	0.662331487312914	0.0	0.5592206790123457	0.0	0.373275893639748	0.0	0.08812651401568204	0.0
ATRX	ATRX chromatin remodeler	0.6556311728395061	0.0	0.6556311728395061	0.0	0.0	0.0	0.0	0.0
HNF1A	HNF1 homeobox A	0.6552025662516333	0.0	0.5939663973414934	0.0	0.0	0.0	0.24494467564055972	0.0
AR	androgen receptor	0.6505240334339668	0.0	0.48055555555555557	0.225	0.5	0.0	0.08616231272124636	0.0
AKT1	AKT serine/threonine kinase 1	0.6486487590891844	0.0	0.5555555555555556	0.2	0.0	0.0	0.2834839252456263	0.0
SMARCA4	SWI/SNF related, matrix associated, actin dependent regulator of chromatin, subfamily a, member 4	0.6472277056260018	0.0	0.5491469478737998	0.0	0.2620701025512506	0.0	0.27584742987491906	0.0
CDKN1A	cyclin dependent kinase inhibitor 1A	0.6356357270112526	0.0	0.3624614197530864	0.0	0.5125359370641707	0.0	0.2923599150792937	0.0
CTNND1	catenin delta 1	0.6337585517736111	0.0	0.3107638888888889	0.0	0.5474765175710412	0.0	0.07731955782312923	0.0

PRKACA	protein kinase cAMP-activated catalytic subunit alpha	0.625	0.0	0.625	0.0	0.0	0.0	0.0	0.0
SETD2	SET domain containing 2, histone lysine methyltransferase	0.6184285314829113	0.0	0.5552083333333334	0.0	0.0	0.0	0.2528807925983116	0.0
DNAJB1	DnaJ heat shock protein family (Hsp40) member B1	0.6139797938061801	0.0	0.5902777777777778	0.0	0.0	0.0061897875384249405	0.09205704742986459	0.0
ERBB3	erb-b2 receptor tyrosine kinase 3	0.6022607315794631	0.0	0.5147707231040565	0.25	0.0	0.0	0.22491007627865964	0.0
TSC1	TSC complex subunit 1	0.5994610138888888	0.0	0.5599392361111111	0.0	0.0	0.0	0.1580871111111111	0.0
HIF1A	hypoxia inducible factor 1 subunit alpha	0.5988293637550938	0.0	0.4814814814814815	0.0	0.32700914244957735	0.0	0.32036036995096107	0.0
MDM2	MDM2 proto-oncogene	0.5971261493852453	0.0	0.4826388888888889	0.0	0.32132713672665114	0.016030888214530907	0.2983819122115699	0.0
TP63	tumor protein p63	0.5965456588544794	0.0	0.4166666666666667	0.0	0.48690800453349176	0.0	0.04923888888888889	0.0
RB1	RB transcriptional corepressor 1	0.595301117069666	0.0	0.5560875896057348	0.0	0.0	0.0	0.1568541098557252	0.0
TBL1XR1	transducin beta like 1 X-linked receptor 1	0.5945910493827161	0.0	0.5861111111111111	0.0	0.0	0.0	0.07631944444444445	0.0
BRCA2	BRCA2 DNA repair associated	0.5944784103249043	0.0	0.5642010381593715	0.0	0.0	0.0	0.12110948866213152	0.0
XPO1	exportin 1	0.5811090256000897	0.0	0.5015432098765432	0.0	0.2787798824737049	0.0	0.08883760594608214	0.0
ARID1B	AT-rich interaction domain 1B	0.5795966392318244	0.0	0.5686299725651577	0.0	0.0	0.0	0.09870000000000001	0.0
TRRAP	transformation/transcription domain associated protein	0.5783380384583254	0.0	0.5128205128205128	0.0	0.2620701025512506	0.0	0.0	0.0
HLA-DQB1	major histocompatibility complex, class II, DQ beta 1	0.5749026631525533	0.558725	0.0	0.0	0.0	0.0	0.06471065261021341	0.0
BLM	BLM RecQ like helicase	0.5740002410698014	0.0	0.5503472222222222	0.0	0.0	0.005067218164946112	0.2100268594104308	0.0
MTOR	mechanistic target of rapamycin kinase	0.5727270781670275	0.0	0.48023504273504275	0.1463611111111111	0.0	0.0	0.30491875901188986	0.0
NOTCH1	notch receptor 1	0.5726441803112589	0.0	0.5268454218106996	0.0	0.0	0.0	0.1831950340022372	0.0
JAK2	Janus kinase 2	0.5723777039170864	0.0	0.4809027777777778	0.1	0.25	0.0	0.2045243352537779	0.0
MICA	MHC class I polypeptide-related sequence A	0.5723063475180985	0.4959176003468815	0.0	0.0	0.0	0.0	0.3055549886848679	0.0
LATS1	large tumor suppressor kinase 1	0.5703243854436789	0.0	0.5183917548500881	0.0	0.0	0.0	0.20773052237436304	0.0
AXIN2	axin 2	0.5695687718501588	0.0	0.5362225651577504	0.0	0.0	0.10098000000000001	0.07291086023167641	0.0
MAGI1	membrane associated guanylate kinase, WW and PDZ domain containing 1	0.5688180827886711	0.0	0.5688180827886711	0.0	0.0	0.0	0.0	0.0
SMAD4	SMAD family member 4	0.5669502894919922	0.0	0.5045438957475995	0.0	0.0	0.0	0.24962557497757104	0.0
WT1	WT1 transcription factor	0.5628203537469423	0.0	0.5038580246913581	0.0	0.0	0.04360038260001573	0.21647136840010772	0.0
LRP1B	LDL receptor related protein 1B	0.5624908315177922	0.0	0.5609908315177923	0.0	0.0	0.0	0.006	0.0
STAT3	signal transducer and activator of transcription 3	0.5616830795949018	0.0	0.48194444444444445	0.0	0.0	0.003823108978598714	0.31725538105578577	0.0
ROS1	ROS proto-oncogene 1, receptor tyrosine kinase	0.5609159069586652	0.0	0.5562114197530865	0.0	0.0	0.01061538485020833	0.014100000000000001	0.0
ALK	ALK receptor tyrosine kinase	0.5607288628065256	0.0	0.5055941358024691	0.0	0.0	0.0	0.22053890801622555	0.0
KMT2A	lysine methyltransferase 2A	0.5592055033383725	0.0	0.541550925925926	0.0	0.0	0.0	0.07061830964978585	0.0
LEF1	lymphoid enhancer binding factor 1	0.5588248547450064	0.0	0.48194444444444445	0.0	0.0	0.09504	0.26528164120224806	0.0
NUP98	nucleoporin 98	0.5586271367521367	0.0	0.5586271367521367	0.0	0.0	0.0	0.0	0.0

BCL2	BCL2 apoptosis regulator	0.558541048059647	0.0	0.4837962962962963	0.0	0.27229961708123734	0.0	0.06002862743737181	0.0
MYC	MYC proto-oncogene, bHLH transcription factor	0.5580777475466973	0.0	0.4837962962962963	0.0	0.0	0.0052287695684767035	0.2948019074156145	0.0
PTPRB	protein tyrosine phosphatase receptor type B	0.5577487863823485	0.0	0.509837962962963	0.0	0.0	0.004597410774469299	0.1896	0.0
SUZ12	SUZ12 polycomb repressive complex 2 subunit	0.5569387304118836	0.0	0.5444315843621399	0.0	0.0	0.0	0.05002858419897464	0.0
ANTXR1	ANTXR cell adhesion molecule 1	0.556795869644154	0.0	0.5336555918663761	0.0	0.0	0.0	0.09256111111111112	0.0
KDM6A	lysine demethylase 6A	0.556287682379349	0.0	0.552587682379349	0.0	0.0	0.0	0.014800000000000002	0.0
NTRK1	neurotrophic receptor tyrosine kinase 1	0.5559287824412941	0.0	0.5	0.2	0.0	0.0	0.05335904197164604	0.0
JAK1	Janus kinase 1	0.5550099773535351	0.0	0.4798280423280423	0.0	0.25	0.0	0.11413741522943456	0.0
NCOR1	nuclear receptor corepressor 1	0.5533784050785039	0.0	0.5459104938271605	0.0	0.0	0.004886201262090616	0.027700000000000002	0.0
FOXA1	forkhead box A1	0.551711981353504	0.0	0.4826388888888889	0.0	0.0	0.0	0.27629236985846023	0.0
ERBB4	erb-b2 receptor tyrosine kinase 4	0.5508286756453423	0.0	0.48042929292929293	0.25	0.0	0.0	0.07109444444444445	0.0
MECOM	MDS1 and EVI1 complex locus	0.5502035881634597	0.0	0.5346628679962013	0.0	0.0	0.0057352315053256775	0.05961388888888889	0.0
SMAD3	SMAD family member 3	0.5476457226977884	0.0	0.48194444444444445	0.0	0.0	0.0	0.2628051130133759	0.0
HNRNPA2B1	heterogeneous nuclear ribonucleoprotein A2/B1	0.5449423981481482	0.0	0.4814814814814815	0.0	0.0	0.0	0.2538436666666667	0.0
AMER1	APC membrane recruitment protein 1	0.5446033950617284	0.0	0.5336033950617284	0.0	0.0	0.0	0.044000000000000004	0.0
KEAP1	kelch like ECH associated protein 1	0.5413374302684604	0.0	0.5334809447128288	0.0	0.0	0.0	0.031425942222526315	0.0
LATS2	large tumor suppressor kinase 2	0.5409468257117662	0.0	0.4814814814814815	0.0	0.0	0.0	0.23786137692113885	0.0
BRD4	bromodomain containing 4	0.5406746153335232	0.0	0.48194444444444445	0.0	0.0	0.0	0.23492068355631493	0.0
DDX3X	DEAD-box helicase 3 X-linked	0.5404599877262534	0.0	0.4837962962962963	0.0	0.0	0.0	0.2266547657198283	0.0
TRIM24	tripartite motif containing 24	0.5393013120265299	0.0	0.4826388888888889	0.0	0.0	0.007975558238768034	0.22310500000000003	0.0
FBXW7	F-box and WD repeat domain containing 7	0.538382195618575	0.0	0.48611111111111116	0.0	0.0	0.0	0.20908433802985535	0.0
FAT4	FAT atypical cadherin 4	0.537964420774739	0.0	0.5219650205761316	0.0	0.0	0.05466426746109662	0.021	0.0
CTCF	CCCTC-binding factor	0.5371270000336295	0.0	0.4807098765432099	0.0	0.0	0.0	0.22566849396167835	0.0
KMT2D	lysine methyltransferase 2D	0.5367155349794239	0.0	0.5367155349794239	0.0	0.0	0.0	0.0	0.0
MYCN	MYCN proto-oncogene, bHLH transcription factor	0.536537962962963	0.0	0.4837962962962963	0.0	0.0	0.0	0.21096666666666666	0.0
SMAD2	SMAD family member 2	0.5345736926932448	0.0	0.5015432098765432	0.0	0.0	0.006318843720557774	0.12931355627989186	0.0
BCORL1	BCL6 corepressor like 1	0.5340701388888889	0.0	0.48055555555555557	0.0	0.0	0.0	0.21405833333333335	0.0
MEN1	menin 1	0.5328018838708323	0.0	0.5126028806584362	0.0	0.0	0.0	0.08079601284958428	0.0
PSMD2	proteasome 26S subunit, non-ATPase 2	0.5325445263227183	0.0	0.1827417695473251	0.1	0.475747972824776	0.0	0.0	0.0
FZR1	fizzy and cell division cycle 20 related 1	0.5324556327160493	0.0	0.5324556327160493	0.0	0.0	0.0	0.0	0.0
AL031847.2	novel transcript	0.5316358024691358	0.0	0.5316358024691358	0.0	0.0	0.0	0.0	0.0
NF2	neurofibromin 2	0.5316305689247922	0.0	0.5086805555555556	0.0	0.0	0.0	0.09180005347694632	0.0
CIC	capicua transcriptional repressor	0.5314521604938272	0.0	0.5146604938271605	0.0	0.0	0.0	0.06716666666666667	0.0
DNMT3A	DNA methyltransferase 3 alpha	0.5304657236665058	0.0	0.4814814814814815	0.0	0.0	0.0	0.1959369687400971	0.0
PRF1	perforin 1	0.5295794444444445	0.0	0.48194444444444445	0.0	0.0	0.0	0.0	0.19054000000000001
EP300	E1A binding protein p300	0.52935229538308	0.0	0.4166666666666667	0.0	0.417319243606814	0.0	0.07079746598639455	0.0
ZFHX3	zinc finger homeobox 3	0.5287114027777777	0.0	0.509765625	0.0	0.0	0.0	0.07578311111111112	0.0

TBX3	T-box 3	0.5280305555555556	0.0	0.5086805555555556	0.0	0.0	0.0	0.07740000000000001	0.0
MAP2K1	mitogen-activated protein kinase kinase 1	0.5245195176514912	0.0	0.4166666666666667	0.2824722222222222	0.25	0.003150151484532672	0.14929618547375884	0.0
BCOR	BCL6 corepressor	0.5244957231040565	0.0	0.5147707231040565	0.0	0.0	0.0	0.0389	0.0
DICER1	dicer 1, ribonuclease III	0.5239339265285772	0.0	0.509548611111111	0.0	0.0	0.012899088757195015	0.05180833333333345	0.0
TET1	tet methylcytosine dioxygenase 1	0.5225722769141387	0.0	0.48611111111111116	0.0	0.0	0.010892276675642798	0.14100365135626883	0.0
MAP3K1	mitogen-activated protein kinase kinase kinase 1	0.5213145833333334	0.0	0.5091145833333334	0.0	0.0	0.0	0.0488	0.0
MYB	MYB proto-oncogene, transcription factor	0.5201984567901234	0.0	0.5026234567901234	0.0	0.0	0.0	0.07030000000000002	0.0
KDM5A	lysine demethylase 5A	0.5199160276441358	0.0	0.5086805555555556	0.0	0.0	0.015775498797221673	0.03793055555555556	0.0
ASXL1	ASXL transcriptional regulator 1	0.5179694919278253	0.0	0.5179694919278253	0.0	0.0	0.0	0.0	0.0
MSH6	mutS homolog 6	0.5175740249625231	0.0	0.505787037037037	0.0	0.0	0.009557891329375588	0.04289999999999994	0.0
MAP2K2	mitogen-activated protein kinase kinase 2	0.5168103055555555	0.0	0.4166666666666667	0.2824722222222222	0.25	0.0	0.027964888888888893	0.0
ERBB2	erb-b2 receptor tyrosine kinase 2	0.5167211187548029	0.0	0.4166666666666667	0.2915	0.0	0.004958456731293913	0.24182593688187395	0.0
CAMTA1	calmodulin binding transcription activator 1	0.5165257936507937	0.0	0.48115079365079366	0.0	0.0	0.0	0.1415	0.0
CLTC	clathrin heavy chain	0.5151932379349046	0.0	0.5087682379349046	0.0	0.0	0.0	0.02570000000000004	0.0
TGFBR2	transforming growth factor beta receptor 2	0.5145411498826575	0.0	0.4166666666666667	0.0	0.3649107375740053	0.0	0.05982118940240548	0.0
SND1	staphylococcal nuclease and tudor domain containing 1	0.5145059631341882	0.0	0.48055555555555557	0.0	0.0	0.0	0.1358016303145306	0.0
MYH9	myosin heavy chain 9	0.5126028806584362	0.0	0.5126028806584362	0.0	0.0	0.0	0.0	0.0
COL2A1	collagen type II alpha 1 chain	0.5122056353070112	0.0	0.509375	0.0	0.0	0.011322541228044951	0.0	0.0
RANBP2	RAN binding protein 2	0.5120876543209877	0.0	0.5084876543209876	0.0	0.0	0.0	0.01440000000000003	0.0
PIK3CB	phosphatidylinositol-4,5-bisphosphate 3-kinase catalytic subunit beta	0.5107222222222222	0.0	0.48194444444444445	0.1	0.0	0.0	0.034	0.0
NCOR2	nuclear receptor corepressor 2	0.5097222222222222	0.0	0.5097222222222222	0.0	0.0	0.0	0.0	0.0
PTPRK	protein tyrosine phosphatase receptor type K	0.5096188271604939	0.0	0.5042438271604939	0.0	0.0	0.0	0.0215	0.0
GPC3	glypican 3	0.5096175403798529	0.0	0.4166666666666667	0.0	0.0	0.11874097684591407	0.31902972736567187	0.0
JAK3	Janus kinase 3	0.5094803591470257	0.0	0.5049803591470258	0.0	0.0	0.0	0.01800000000000002	0.0
CACNA1D	calcium voltage-gated channel subunit alpha1 D	0.5091145833333334	0.0	0.5091145833333334	0.0	0.0	0.0	0.0	0.0
SMOX	spermine oxidase	0.5050592116280685	0.0	0.4807098765432099	0.0	0.0	0.013269015763727505	0.0915	0.0
PTPN13	protein tyrosine phosphatase non-receptor type 13	0.5050426116235932	0.0	0.4800925925925926	0.0	0.0	0.03496745444227047	0.08425898526077098	0.0
FLCN	folliculin	0.5045432098765432	0.0	0.5015432098765432	0.0	0.0	0.0	0.012	0.0
MSH2	mutS homolog 2	0.5045118905828747	0.0	0.48194444444444445	0.0	0.0	0.011729374590504852	0.08505672918016326	0.0
WRN	Werner syndrome RecQ like helicase	0.5016212962962963	0.0	0.4837962962962963	0.0	0.0	0.0	0.0713	0.0
SFRP4	secreted frizzled related protein 4	0.5009076574074074	0.0	0.4837962962962963	0.0	0.0	0.0	0.06844544444444445	0.0
ERCC3	ERCC excision repair 3, TFIIH core complex helicase subunit	0.49878682407407404	0.0	0.4837962962962963	0.0	0.0	0.0	0.05996211111111111	0.0
BRCA1	BRCA1 DNA repair associated	0.49876626524641393	0.0	0.48194444444444445	0.0	0.0	0.0	0.06728728320787793	0.0
CALR	calreticulin	0.49852464353336484	0.0	0.4837962962962963	0.0	0.0	0.013533906293537094	0.05289831948448008	0.0
POLQ	DNA polymerase theta	0.4980527777777778	0.0	0.4809027777777778	0.0	0.0	0.0686	0.0	0.0
DNM2	dynamin 2	0.4978462962962963	0.0	0.4837962962962963	0.0	0.0	0.0	0.05620000000000001	0.0
ACVR1	activin A receptor type 1	0.4964737962962963	0.0	0.4837962962962963	0.0	0.0	0.0	0.05071000000000005	0.0

AKT2	AKT serine/threonine kinase 2	0.49611769391160027	0.0	0.4166666666666667	0.2	0.0	0.0	0.2289152200908456	0.0
TET2	tet methylcytosine dioxygenase 2	0.4961064514898059	0.0	0.4166666666666667	0.0	0.0	0.0	0.17906431340825282	0.23817500000000003
CXCR4	C-X-C motif chemokine receptor 4	0.49468317542109247	0.0	0.4166666666666667	0.0	0.0	0.0	0.31206603501770314	0.0
SALL4	spalt like transcription factor 4	0.4932813293571418	0.0	0.4166666666666667	0.0	0.0	0.0	0.3064586507619005	0.0
MALT1	MALT1 paracaspase	0.492425	0.0	0.48194444444444445	0.0	0.0	0.0	0.04192222222222222	0.0
NCOA2	nuclear receptor coactivator 2	0.4913231481481482	0.0	0.4814814814814815	0.0	0.0	0.0	0.03936666666666667	0.0
ABL2	ABL proto-oncogene 2, non-receptor tyrosine kinase	0.49111111111111116	0.0	0.48611111111111116	0.0	0.0	0.0	0.020000000000000004	0.0
STAG2	stromal antigen 2	0.4908962962962963	0.0	0.4837962962962963	0.0	0.0	0.0	0.0284	0.0
CBL	Cbl proto-oncogene	0.49081216312193915	0.0	0.4166666666666667	0.0	0.29658198582108986	0.0	0.0	0.0
ACKR3	atypical chemokine receptor 3	0.4904848443977607	0.0	0.4166666666666667	0.0	0.0	0.006644202271681783	0.29231973213696205	0.0
KDM5C	lysine demethylase 5C	0.49032929292929295	0.0	0.48042929292929293	0.0	0.0	0.0	0.0396	0.0
IKZF1	IKAROS family zinc finger 1	0.4898157088325562	0.0	0.4809027777777778	0.0	0.0	0.01631637949300601	0.028399999999999998	0.0
IL7R	interleukin 7 receptor	0.4897674625239779	0.0	0.4826388888888889	0.0	0.0	0.006075912715801404	0.025813888888888893	0.0
KLF6	Kruppel like factor 6	0.4896506742959838	0.0	0.4166666666666667	0.0	0.0	0.0	0.2919360305172685	0.0
PPP6C	protein phosphatase 6 catalytic subunit	0.4892962962962963	0.0	0.4837962962962963	0.0	0.0	0.0	0.022000000000000002	0.0
KLF4	Kruppel like factor 4	0.4892917721325513	0.0	0.4166666666666667	0.0	0.0	0.01105020195706419	0.2855892209937323	0.0
SH2B3	SH2B adaptor protein 3	0.4884234241560314	0.0	0.48194444444444445	0.0	0.0	0.013310817404282369	0.020000000000000004	0.0
ERCC4	ERCC excision repair 4, endonuclease catalytic subunit	0.4883992610503722	0.0	0.48115079365079366	0.0	0.0	0.01843620659620696	0.020800000000000003	0.0
SDHA	succinate dehydrogenase complex flavoprotein subunit A	0.4877814814814815	0.0	0.4814814814814815	0.0	0.0	0.0	0.0252	0.0
TLX3	T cell leukemia homeobox 3	0.4876388888888889	0.0	0.4826388888888889	0.0	0.0	0.0	0.020000000000000004	0.0
ETNK1	ethanolamine kinase 1	0.4873492581130266	0.0	0.4826388888888889	0.0	0.0	0.018841476896550798	0.0	0.0
UBR5	ubiquitin protein ligase E3 component n-recognin 5	0.4870277777777778	0.0	0.4809027777777778	0.0	0.0	0.0	0.024500000000000004	0.0
MTUS1	microtubule associated scaffold protein 1	0.48688888888888887	0.47638888888888886	0.0	0.0	0.0	0.0	0.042	0.0
KAT6A	lysine acetyltransferase 6A	0.4858555555555556	0.0	0.48055555555555557	0.0	0.0	0.0	0.021200000000000004	0.0
EPS15	epidermal growth factor receptor pathway substrate 15	0.48564444444444443	0.0	0.48194444444444445	0.0	0.0	0.0	0.014800000000000002	0.0
MED12	mediator complex subunit 12	0.48475079365079365	0.0	0.48115079365079366	0.0	0.0	0.0	0.014400000000000003	0.0
ZNF331	zinc finger protein 331	0.484495976017878	0.0	0.4814814814814815	0.0	0.0	0.01205797814558599	0.0	0.0
AFDN	afadin, adherens junction formation factor	0.4841388888888889	0.0	0.4826388888888889	0.0	0.0	0.0	0.006	0.0
ZRSR2	zinc finger CCCH-type, RNA binding motif and serine/arginine rich 2	0.4837962962962963	0.0	0.4837962962962963	0.0	0.0	0.0	0.0	0.0
TAL1	TAL bHLH transcription factor 1, erythroid differentiation factor	0.4837962962962963	0.0	0.4837962962962963	0.0	0.0	0.0	0.0	0.0
IRF4	interferon regulatory factor 4	0.4837962962962963	0.0	0.4837962962962963	0.0	0.0	0.0	0.0	0.0
MLLT10	MLLT10 histone lysine methyltransferas	0.4837962962962963	0.0	0.4837962962962963	0.0	0.0	0.0	0.0	0.0

	e DOT1L cofactor								
HOXA11	homeobox A11	0.4837350885164541	0.0	0.4166666666666667	0.0	0.0	0.017242796648086516	0.26061022222222224	0.0
IKBKB	inhibitor of nuclear factor kappa B kinase subunit beta	0.4836152235054762	0.0	0.4166666666666667	0.0	0.0	0.0	0.26779422735523806	0.0
PML	promyelocytic leukemia	0.4832134898397811	0.0	0.4166666666666667	0.0	0.0	0.0	0.2661872926924575	0.0
NOTCH2	notch receptor 2	0.48302844104243325	0.0	0.4166666666666667	0.0	0.0	0.0	0.26544709750306617	0.0
BCL11B	BAF chromatin remodeling complex subunit BCL11B	0.4826388888888889	0.0	0.4826388888888889	0.0	0.0	0.0	0.0	0.0
EIF3E	eukaryotic translation initiation factor 3 subunit E	0.4826388888888889	0.0	0.4826388888888889	0.0	0.0	0.0	0.0	0.0
NDRG1	N-myc downstream regulated 1	0.4820581214881947	0.0	0.4166666666666667	0.0	0.0	0.0	0.2615658192861121	0.0
ERG	ETS transcription factor ERG	0.48194444444444445	0.0	0.48194444444444445	0.0	0.0	0.0	0.0	0.0
PRDM1	PR/SET domain 1	0.48169248166210565	0.0	0.4166666666666667	0.0	0.25370325998175586	0.0	0.014400000000000003	0.0
BCL9	BCL9 transcription coactivator	0.48152770266677103	0.0	0.4166666666666667	0.0	0.0	0.005073477062163406	0.25718926530612246	0.0
BUB1B	BUB1 mitotic checkpoint serine/threonine kinase B	0.48151342943681436	0.0	0.4166666666666667	0.0	0.0	0.10493041777408385	0.21275130984766458	0.0
MRPL36	mitochondrial ribosomal protein L36	0.4814814814814815	0.0	0.4814814814814815	0.0	0.0	0.0	0.0	0.0
AFF3	AF4/FMR2 family member 3	0.4814814814814815	0.0	0.4814814814814815	0.0	0.0	0.0	0.0	0.0
USP6	ubiquitin specific peptidase 6	0.4814814814814815	0.0	0.4814814814814815	0.0	0.0	0.0	0.0	0.0
ATP2B3	ATPase plasma membrane Ca2+ transporting 3	0.4814814814814815	0.0	0.4814814814814815	0.0	0.0	0.0	0.0	0.0
ERCC2	ERCC excision repair 2, TFIIH core complex helicase subunit	0.4814439823628123	0.0	0.4166666666666667	0.0	0.0	0.0	0.25910926278458246	0.0
CEBPA	CCAAT enhancer binding protein alpha	0.4814352989259465	0.0	0.4166666666666667	0.0	0.0	0.0	0.1542026903335187	0.19054000000000001
GRIN2A	glutamate ionotropic receptor NMDA type subunit 2A	0.48115079365079366	0.0	0.48115079365079366	0.0	0.0	0.0	0.0	0.0
CLTCL1	clathrin heavy chain like 1	0.48115079365079366	0.0	0.48115079365079366	0.0	0.0	0.0	0.0	0.0
FANCA	FA complementation group A	0.4809027777777778	0.0	0.4809027777777778	0.0	0.0	0.0	0.0	0.0
PTPN11	protein tyrosine phosphatase non-receptor type 11	0.4807721850516147	0.0	0.4166666666666667	0.0	0.0	0.0	0.2564220735397921	0.0
CDK12	cyclin dependent kinase 12	0.4807098765432099	0.0	0.4807098765432099	0.0	0.0	0.0	0.0	0.0
BCL11A	BAF chromatin remodeling complex subunit BCL11A	0.4807098765432099	0.0	0.4807098765432099	0.0	0.0	0.0	0.0	0.0
EPAS1	endothelial PAS domain protein 1	0.48062847796089736	0.0	0.4166666666666667	0.0	0.0	0.0	0.2558472451769226	0.0
STAT4	signal transducer and activator of transcription 4	0.4805076475706178	0.42028870018789416	0.0	0.0	0.0	0.0	0.2408757895308946	0.0
CARD11	caspase recruitment domain family member 11	0.48032407407407407	0.0	0.48032407407407407	0.0	0.0	0.0	0.0	0.0
SPEN	spen family transcriptional repressor	0.4800925925925926	0.0	0.4800925925925926	0.0	0.0	0.0	0.0	0.0
ZNF521	zinc finger protein 521	0.4800347222222222	0.0	0.4800347222222222	0.0	0.0	0.0	0.0	0.0
BCL6	BCL6 transcription repressor	0.4785326388888889	0.0	0.4166666666666667	0.0	0.0	0.0	0.020900000000000005	0.23817500000000003
TPM3	tropomyosin 3	0.47825420154357384	0.0	0.4166666666666667	0.0	0.0	0.010682813892164526	0.24160222222222225	0.0
LCK	LCK proto-oncogene, Src family tyrosine kinase	0.47795000000000004	0.0	0.4166666666666667	0.2361111111111111	0.0	0.0	0.020300000000000002	0.0

PTPRC	protein tyrosine phosphatase receptor type C	0.4772346951332031	0.0	0.4166666666666667	0.0	0.0	0.007787540807571794	0.11201676449456875	0.19054000000000001
CYLD	CYLD lysine 63 deubiquitinase	0.47671678165773884	0.0	0.4166666666666667	0.0	0.0	0.009640183900360177	0.23591593378635073	0.0
FOXP1	forkhead box P1	0.47436292830372895	0.0	0.4166666666666667	0.0	0.0	0.0	0.23078504654824894	0.0
ATP1A1	ATPase Na+/K+ transporting subunit alpha 1	0.4715894529478458	0.0	0.4166666666666667	0.0	0.0	0.0	0.21969114512471655	0.0
PPM1D	protein phosphatase, Mg2+/Mn2+ dependent 1D	0.47124533333333335	0.0	0.4166666666666667	0.0	0.0	0.0	0.21831466666666668	0.0
EXT1	exostosin glycosyltransferase 1	0.4696368055555556	0.0	0.4166666666666667	0.0	0.0	0.0	0.21188055555555557	0.0
CDKN3	cyclin dependent kinase inhibitor 3	0.46949039181922636	0.4375	0.0	0.0	0.0	0.11009682653616469	0.040195666666666664	0.0
SDHB	succinate dehydrogenase complex iron sulfur subunit B	0.46842500000000004	0.0	0.4166666666666667	0.0	0.0	0.0	0.20703333333333335	0.0
TNFAIP3	TNF alpha induced protein 3	0.46839872839506175	0.0	0.4166666666666667	0.0	0.0	0.0	0.036873555555555555	0.19054000000000001
RECQL4	RecQ like helicase 4	0.468275	0.0	0.4166666666666667	0.0	0.0	0.0	0.20643333333333336	0.0
BARD1	BRCA1 associated RING domain 1	0.4682158201960016	0.0	0.4166666666666667	0.0	0.0	0.01394238176401465	0.2	0.0
MRTFA	myocardin related transcription factor A	0.46412083333333337	0.0	0.4166666666666667	0.0	0.0	0.0	0.18981666666666666	0.0
STIL	STIL centriolar assembly protein	0.46376825127417776	0.0	0.4166666666666667	0.0	0.0	0.013064261467599642	0.1826	0.0
BIRC3	baculoviral IAP repeat containing 3	0.45849160260770977	0.0	0.4166666666666667	0.0	0.0	0.0	0.16729974376417228	0.0
MLH1	mutL homolog 1	0.45577092743764175	0.0	0.4166666666666667	0.0	0.0	0.0	0.15641704308390023	0.0
VHL	von Hippel-Lindau tumor suppressor	0.45462545607278726	0.0	0.4166666666666667	0.0	0.0	0.0	0.15183515762448235	0.0
SOX2	SRY-box 2	0.4545158407071012	0.0	0.4166666666666667	0.0	0.0	0.0	0.1513966961617381	0.0
PIK3R1	phosphoinositide-3-kinase regulatory subunit 1	0.4542739086356765	0.0	0.4166666666666667	0.1	0.0	0.0	0.10598452343159487	0.0
PDCD1LG2	programmed cell death 1 ligand 2	0.4524387940759637	0.0	0.4166666666666667	0.0	0.0	0.0	0.1430885096371882	0.0
XPC	XPC complex subunit, DNA damage recognition and repair factor	0.45214529662698416	0.0	0.4166666666666667	0.0	0.0	0.0	0.14191451984126982	0.0
SSX1	SSX family member 1	0.451819054414424	0.0	0.4166666666666667	0.0	0.0	0.1308762176576958	0.021900000000000003	0.0
PLAG1	PLAG1 zinc finger	0.4508166666666667	0.0	0.4166666666666667	0.0	0.0	0.0	0.1366	0.0
GATA2	GATA binding protein 2	0.44998125	0.0	0.4166666666666667	0.0	0.0	0.0	0.1332583333333333	0.0
CTNNA2	catenin alpha 2	0.4467091070046468	0.0	0.3100198412698413	0.0	0.3566691467290076	0.10494000000000002	0.014000000000000002	0.0
DDX5	DEAD-box helicase 5	0.4466041666666667	0.0	0.4166666666666667	0.0	0.0	0.0	0.11975000000000001	0.0
WIF1	WNT inhibitory factor 1	0.44486060182854975	0.0	0.4166666666666667	0.0	0.0	0.0	0.1127757406475322	0.0
PLCG1	phospholipase C gamma 1	0.44374366383219954	0.0	0.4166666666666667	0.0	0.0	0.0	0.10830798866213152	0.0
FBXO11	F-box protein 11	0.44156666666666666	0.0	0.4166666666666667	0.0	0.0	0.0	0.09960000000000001	0.0
PPP2R1A	protein phosphatase 2 scaffold subunit Aalpha	0.44106666666666666	0.0	0.4166666666666667	0.0	0.0	0.0	0.0976	0.0
FAT1	FAT atypical cadherin 1	0.4409659722222222	0.0	0.4166666666666667	0.0	0.0	0.0	0.09719722222222224	0.0
SYK	spleen associated tyrosine kinase	0.440836114312173	0.0	0.4166666666666667	0.0	0.0	0.0	0.09667779058202529	0.0
STAT6	signal transducer and activator of transcription 6	0.4389903825113379	0.0	0.4166666666666667	0.0	0.0	0.0	0.08929486337868481	0.0
PREX2	phosphatidylinositol-3,4,5-trisphosphate dependent Rac exchange factor 2	0.4388004166666667	0.0	0.4166666666666667	0.0	0.0	0.0	0.088535	0.0
NTRK3	neurotrophic receptor tyrosine kinase 3	0.4387444444444445	0.0	0.4166666666666667	0.0	0.0	0.0	0.08831111111111113	0.0

SLC34A2	solute carrier family 34 member 2	0.43868244846000704	0.0	0.4166666666666667	0.0	0.0	0.05819203614006318	0.062200000000000005	0.0
SRSF2	serine and arginine rich splicing factor 2	0.4383881859410431	0.0	0.4166666666666667	0.0	0.0	0.0	0.08688607709750566	0.0
FUS	FUS RNA binding protein	0.438197002227344	0.0	0.4166666666666667	0.0	0.0	0.0070652215767075905	0.08298124376417236	0.0
SPOP	speckle type BTB/POZ protein	0.43801666666666667	0.0	0.4166666666666667	0.0	0.0	0.0	0.0854	0.0
RARA	retinoic acid receptor alpha	0.4374992716648472	0.0	0.4166666666666667	0.0	0.0	0.0	0.08333041999272202	0.0
TRIM33	tripartite motif containing 33	0.43735625	0.0	0.4166666666666667	0.0	0.0	0.0	0.08275833333333334	0.0
FANCD2	FA complementation group D2	0.4357686171263715	0.0	0.4166666666666667	0.0	0.0	0.016173804137342988	0.06921944444444444	0.0
ZBTB16	zinc finger and BTB domain containing 16	0.43560347222222223	0.0	0.4166666666666667	0.0	0.0	0.0	0.07574722222222222	0.0
BCL3	BCL3 transcription coactivator	0.43556666666666666	0.0	0.4166666666666667	0.0	0.0	0.0	0.0756	0.0
PTCH1	patched 1	0.43525435709886956	0.0	0.4166666666666667	0.0	0.0	0.0	0.07435076172881151	0.0
CHEK2	checkpoint kinase 2	0.4352194265103698	0.0	0.4166666666666667	0.0	0.0	0.013346607867704492	0.06827921365583273	0.0
FANCG	FA complementation group G	0.43431935855530024	0.0	0.4166666666666667	0.0	0.0	0.01824922699770185	0.0625	0.0
NRG1	neuregulin 1	0.43396308152409213	0.0	0.4166666666666667	0.0	0.0	0.013530233716829067	0.06317222222222223	0.0
MUTYH	mutY DNA glycosylase	0.43350864002267575	0.0	0.4166666666666667	0.0	0.0	0.0	0.06736789342403629	0.0
PAX5	paired box 5	0.43350493367346943	0.0	0.4166666666666667	0.0	0.0	0.0	0.06735306802721089	0.0
NFATC2	nuclear factor of activated T cells 2	0.4328423055555556	0.0	0.4166666666666667	0.0	0.0	0.0	0.06470255555555557	0.0
ETV4	ETS variant 4	0.43251319444444447	0.0	0.4166666666666667	0.0	0.0	0.0	0.06338611111111112	0.0
BAX	BCL2 associated X, apoptosis regulator	0.4323517138778767	0.0	0.4166666666666667	0.0	0.0	0.0	0.06274018884484003	0.0
DCC	DCC netrin 1 receptor	0.4322243696882377	0.0	0.32187273057371096	0.0	0.3422933889124869	0.0	0.08516518319090724	0.0
CHD4	chromodomain helicase DNA binding protein 4	0.4322166666666667	0.0	0.4166666666666667	0.0	0.0	0.0	0.062200000000000005	0.0
NSD2	nuclear receptor binding SET domain protein 2	0.4320666666666667	0.0	0.4166666666666667	0.0	0.0	0.0	0.0616	0.0
DDIT3	DNA damage inducible transcript 3	0.4320599676819175	0.0	0.4166666666666667	0.0	0.0	0.0	0.06157320406100331	0.0
ERCC5	ERCC excision repair 5, endonuclease	0.431687612244898	0.0	0.4166666666666667	0.0	0.0	0.0	0.06008378231292517	0.0
CBFA2T3	CBFA2/RUNX1 translocation partner 3	0.43116448221260667	0.0	0.4166666666666667	0.0	0.0	0.05159126218376003	0.014400000000000003	0.0
PTPRT	protein tyrosine phosphatase receptor type T	0.4297472222222222	0.0	0.4166666666666667	0.0	0.0	0.0	0.05232222222222223	0.0
DROSHA	drosha ribonuclease III	0.4296976730850898	0.0	0.4166666666666667	0.0	0.0	0.003879057765808491	0.0504	0.0
BTK	Bruton tyrosine kinase	0.42872675000000005	0.0	0.4166666666666667	0.0	0.0	0.0	0.048240333333333336	0.0
PBX1	PBX homeobox 1	0.42871192057478624	0.0	0.4166666666666667	0.0	0.0	0.048181015632478126	0.0	0.0
SRSF3	serine and arginine rich splicing factor 3	0.4286416666666667	0.0	0.4166666666666667	0.0	0.0	0.0	0.0479	0.0
FOXO4	forkhead box O4	0.4286183333333334	0.0	0.4166666666666667	0.0	0.0	0.0	0.04780666666666667	0.0
NKX2-1	NK2 homeobox 1	0.42769722222222223	0.0	0.4166666666666667	0.0	0.0	0.0	0.04412222222222222	0.0
SMARCB1	SWI/SNF related, matrix associated, actin dependent regulator of chromatin, subfamily b, member 1	0.42765000000000003	0.0	0.4166666666666667	0.0	0.0	0.0	0.043933333333333345	0.0
PMS2	PMS1 homolog 2, mismatch repair system component	0.4274916666666667	0.0	0.4166666666666667	0.0	0.0	0.0	0.043300000000000005	0.0
RUNX1T1	RUNX1 translocation partner 1	0.42646666666666666	0.0	0.4166666666666667	0.0	0.0	0.0	0.039200000000000006	0.0
RNF43	ring finger protein 43	0.4264205833333333	0.0	0.4166666666666667	0.0	0.0	0.0	0.03901566666666667	0.0
RUNX1	RUNX family transcription factor 1	0.4261666666666667	0.0	0.4166666666666667	0.0	0.0	0.0	0.038	0.0

CREB3L1	cAMP responsive element binding protein 3 like 1	0.4250666666666667	0.0	0.4166666666666667	0.0	0.0	0.0	0.0336	0.0
CDH11	cadherin 11	0.42496666666666666	0.0	0.4166666666666667	0.0	0.0	0.0	0.0332	0.0
ELK4	ETS transcription factor ELK4	0.4245666666666667	0.0	0.4166666666666667	0.0	0.0	0.0	0.0316	0.0
TCF7L1	transcription factor 7 like 1	0.4242666666666667	0.0	0.4166666666666667	0.0	0.0	0.0	0.0304	0.0
POT1	protection of telomeres 1	0.42378027777777777	0.0	0.4166666666666667	0.0	0.0	0.0	0.028454444444444445	0.0
WAS	WASP actin nucleation promoting factor	0.4237666666666667	0.0	0.4166666666666667	0.0	0.0	0.0	0.028399999999999998	0.0
PAX3	paired box 3	0.42341977479777054	0.0	0.4166666666666667	0.0	0.0	0.023990210302193272	0.0068000000000000005	0.0
BMPR1A	bone morphogenetic protein receptor type 1A	0.42305833333333337	0.0	0.4166666666666667	0.0	0.0	0.0	0.025566666666666668	0.0
RAD21	RAD21 cohesin complex component	0.4230085491339976	0.0	0.4166666666666667	0.0	0.0	0.003976942205977979	0.023600000000000003	0.0
RBM10	RNA binding motif protein 10	0.4228525	0.0	0.4166666666666667	0.0	0.0	0.0	0.024743333333333336	0.0
NBN	nibrin	0.42278362736223	0.0	0.4166666666666667	0.0	0.0	0.004527646260070069	0.022455555555555556	0.0
LRIG3	leucine rich repeats and immunoglobulin like domains 3	0.4227666666666667	0.0	0.4166666666666667	0.0	0.0	0.0	0.024400000000000005	0.0
DDX6	DEAD-box helicase 6	0.4226916666666667	0.0	0.4166666666666667	0.0	0.0	0.0	0.024100000000000003	0.0
SETBP1	SET binding protein 1	0.42241666666666666	0.0	0.4166666666666667	0.0	0.0	0.0	0.023000000000000003	0.0
EWSR1	EWS RNA binding protein 1	0.4222777777777778	0.0	0.4166666666666667	0.0	0.0	0.0	0.022444444444444445	0.0
EBF1	EBF transcription factor 1	0.42220437980103	0.0	0.4166666666666667	0.0	0.0	0.004839418209269565	0.020000000000000004	0.0
HIST1H3B	histone cluster 1 H3 family member b	0.42161455233487527	0.0	0.4166666666666667	0.0	0.0	0.01979154267283437	0.0	0.0
MYCL	MYCL proto-oncogene, bHLH transcription factor	0.42134591658290177	0.0	0.4166666666666667	0.0	0.0	0.008813249246116114	0.014800000000000002	0.0
RHOH	ras homolog family member H	0.4211666666666667	0.0	0.4166666666666667	0.0	0.0	0.0	0.018000000000000002	0.0
RAD51B	RAD51 paralog B	0.4210666666666667	0.0	0.4166666666666667	0.0	0.0	0.0	0.0176	0.0
NSD3	nuclear receptor binding SET domain protein 3	0.4209666666666667	0.0	0.4166666666666667	0.0	0.0	0.0	0.0172	0.0
NR4A3	nuclear receptor subfamily 4 group A member 3	0.4208994168258737	0.0	0.4166666666666667	0.0	0.0	0.005694751432862773	0.014400000000000003	0.0
LMO2	LIM domain only 2	0.42086666666666667	0.0	0.4166666666666667	0.0	0.0	0.0	0.016800000000000002	0.0
LPP	LIM domain containing preferred translocation partner in lipoma	0.4206666666666667	0.0	0.4166666666666667	0.0	0.0	0.0	0.016	0.0
SMARCD1	SWI/SNF related, matrix associated, actin dependent regulator of chromatin, subfamily d, member 1	0.4206666666666667	0.0	0.4166666666666667	0.0	0.0	0.0	0.016	0.0
IRS4	insulin receptor substrate 4	0.4205666666666667	0.0	0.4166666666666667	0.0	0.0	0.0	0.015600000000000003	0.0
MMAB	metabolism of cobalamin associated B	0.42049914302850694	0.0	0.4166666666666667	0.0	0.0	0.015329905447361042	0.0	0.0
MAP3K13	mitogen-activated protein kinase kinase kinase 13	0.42036666666666667	0.0	0.4166666666666667	0.0	0.0	0.0	0.014800000000000002	0.0
REL	REL proto-oncogene, NF-kB subunit	0.4202666666666667	0.0	0.4166666666666667	0.0	0.0	0.0	0.014400000000000003	0.0
MYOD1	myogenic differentiation 1	0.4202666666666667	0.0	0.4166666666666667	0.0	0.0	0.0	0.014400000000000003	0.0
SUFU	SUFU negative regulator of hedgehog signaling	0.4202666666666667	0.0	0.4166666666666667	0.0	0.0	0.0	0.014400000000000003	0.0
CARS	cysteinyl-tRNA synthetase	0.41987475465658874	0.0	0.4166666666666667	0.0	0.0	0.012832351959688152	0.0	0.0
ETV6	ETS variant 6	0.41941666666666666	0.0	0.4166666666666667	0.0	0.0	0.0	0.011000000000000003	0.0

MN1	MN1 proto-oncogene, transcriptional regulator	0.41938157635073525	0.0	0.4166666666666667	0.0	0.0	0.01085963873627426	0.0	0.0
ELF4	E74 like ETS transcription factor 4	0.4186666666666667	0.0	0.4166666666666667	0.0	0.0	0.0	0.008	0.0
KNL1	kinetochore scaffold 1	0.41808427412237875	0.0	0.4166666666666667	0.0	0.0	0.005670429822848301	0.0	0.0
CUX1	cut like homeobox 1	0.4166666666666667	0.0	0.4166666666666667	0.0	0.0	0.0	0.0	0.0
AP003108.2	novel transcript	0.4166666666666667	0.0	0.4166666666666667	0.0	0.0	0.0	0.0	0.0
ARHGEF12	Rho guanine nucleotide exchange factor 12	0.4166666666666667	0.0	0.4166666666666667	0.0	0.0	0.0	0.0	0.0
MITF	melanocyte inducing transcription factor	0.4166666666666667	0.0	0.4166666666666667	0.0	0.0	0.0	0.0	0.0
BCL9L	BCL9 like	0.4166666666666667	0.0	0.4166666666666667	0.0	0.0	0.0	0.0	0.0
MAML2	mastermind like transcriptional coactivator 2	0.4166666666666667	0.0	0.4166666666666667	0.0	0.0	0.0	0.0	0.0
DDX10	DEAD-box helicase 10	0.4166666666666667	0.0	0.4166666666666667	0.0	0.0	0.0	0.0	0.0
MLF1	myeloid leukemia factor 1	0.4166666666666667	0.0	0.4166666666666667	0.0	0.0	0.0	0.0	0.0
BRD3	bromodomain containing 3	0.4166666666666667	0.0	0.4166666666666667	0.0	0.0	0.0	0.0	0.0
FEV	FEV transcription factor, ETS family member	0.4166666666666667	0.0	0.4166666666666667	0.0	0.0	0.0	0.0	0.0
PGAP3	post-GPI attachment to proteins 3	0.4166666666666667	0.0	0.4166666666666667	0.0	0.0	0.0	0.0	0.0
KAT6B	lysine acetyltransferase 6B	0.4166666666666667	0.0	0.4166666666666667	0.0	0.0	0.0	0.0	0.0
FLI1	Fli-1 proto-oncogene, ETS transcription factor	0.4166666666666667	0.0	0.4166666666666667	0.0	0.0	0.0	0.0	0.0
EXT2	exostosin glycosyltransferase 2	0.4166666666666667	0.0	0.4166666666666667	0.0	0.0	0.0	0.0	0.0
PRDM16	PR/SET domain 16	0.4166666666666667	0.0	0.4166666666666667	0.0	0.0	0.0	0.0	0.0
CBLC	Cbl proto-oncogene C	0.4166666666666667	0.0	0.4166666666666667	0.0	0.0	0.0	0.0	0.0
SH3GL1	SH3 domain containing GRB2 like 1, endophilin A2	0.4166666666666667	0.0	0.4166666666666667	0.0	0.0	0.0	0.0	0.0
RAP1GDS1	Rap1 GTPase-GDP dissociation stimulator 1	0.4166666666666667	0.0	0.4166666666666667	0.0	0.0	0.0	0.0	0.0
USP8	ubiquitin specific peptidase 8	0.4166666666666667	0.0	0.4166666666666667	0.0	0.0	0.0	0.0	0.0
CDC73	cell division cycle 73	0.4166666666666667	0.0	0.4166666666666667	0.0	0.0	0.0	0.0	0.0
TRAF7	TNF receptor associated factor 7	0.4166666666666667	0.0	0.4166666666666667	0.0	0.0	0.0	0.0	0.0
HIP1	huntingtin interacting protein 1	0.4166666666666667	0.0	0.4166666666666667	0.0	0.0	0.0	0.0	0.0
RPL5	ribosomal protein L5	0.4166666666666667	0.0	0.4166666666666667	0.0	0.0	0.0	0.0	0.0
KCNJ5	potassium voltage-gated channel subfamily J member 5	0.4166666666666667	0.0	0.4166666666666667	0.0	0.0	0.0	0.0	0.0
FANCE	FA complementation group E	0.4166666666666667	0.0	0.4166666666666667	0.0	0.0	0.0	0.0	0.0
CCDC6	coiled-coil domain containing 6	0.4166666666666667	0.0	0.4166666666666667	0.0	0.0	0.0	0.0	0.0
ELL	elongation factor for RNA polymerase II	0.4166666666666667	0.0	0.4166666666666667	0.0	0.0	0.0	0.0	0.0
LZTR1	leucine zipper like transcription regulator 1	0.4166666666666667	0.0	0.4166666666666667	0.0	0.0	0.0	0.0	0.0
FH	fumarate hydratase	0.4166666666666667	0.0	0.4166666666666667	0.0	0.0	0.0	0.0	0.0
CNOT3	CCR4-NOT transcription complex subunit 3	0.4166666666666667	0.0	0.4166666666666667	0.0	0.0	0.0	0.0	0.0

PALB2	partner and localizer of BRCA2	0.4166666666666667	0.0	0.4166666666666667	0.0	0.0	0.0	0.0	0.0
TFE3	transcription factor binding to IGHM enhancer 3	0.4166666666666667	0.0	0.4166666666666667	0.0	0.0	0.0	0.0	0.0
CD79B	CD79b molecule	0.4166666666666667	0.0	0.4166666666666667	0.0	0.0	0.0	0.0	0.0
CDK14	cyclin dependent kinase 14	0.4138900470595945	0.39198282459096256	0.0	0.0	0.0	0.0	0.08762888987452773	0.0
DEK	DEK proto-oncogene	0.41305078008334406	0.0	0.3472222222222222	0.0	0.0	0.0	0.2633142314444875	0.0
MACC1	MET transcriptional regulator MACC1	0.41246662207668994	0.0	0.33984375	0.0	0.0	0.0028969807138033137	0.28920394132284705	0.0
STK11	serine/threonine kinase 11	0.41190609981119336	0.0	0.3472222222222222	0.0	0.0	0.0	0.25873551035588455	0.0
GRIK1	glutamate ionotropic receptor kainate type subunit 1	0.4089696331454392	0.4030446331454392	0.0	0.0	0.0	0.0	0.023700000000000006	0.0
SIX1	SIX homeobox 1	0.40842862108676387	0.0	0.3472222222222222	0.0	0.0	0.0247510540665891	0.233825126984127	0.0
CYP2C8	cytochrome P450 family 2 subfamily C member 8	0.40232488314324594	0.0	0.33912037037037035	0.0	0.0	0.11485360985383936	0.20177200226757372	0.0
MUC16	mucin 16, cell surface associated	0.40181998824354903	0.0	0.38160540591096154	0.0	0.0	0.0	0.08085832933035005	0.0
PNPLA3	patatin like phospholipase domain containing 3	0.40125906192215466	0.3336220335212275	0.0	0.0	0.0	0.015858123910798407	0.2635000585322426	0.0
SETDB1	SET domain bifurcated histone lysine methyltransferase 1	0.39879582095616023	0.0	0.35320216049382713	0.0	0.0	0.0	0.25953513567649283	0.0
LIFR	LIF receptor alpha	0.3903583270933104	0.0	0.33940972222222227	0.0	0.0	0.06269244948447719	0.1759311086023626	0.0
PPARG	peroxisome proliferator activated receptor gamma	0.38088171614561983	0.0	0.2777777777777778	0.0	0.0	0.0	0.3114372717011754	0.0
ARHGAP5	Rho GTPase activating protein 5	0.38043117283950617	0.0	0.3395061728395062	0.0	0.0	0.0	0.1637	0.0
CDKN1B	cyclin dependent kinase inhibitor 1B	0.37990430927026553	0.0	0.3472222222222222	0.0	0.0	0.0	0.1307283481921732	0.0
HOXA13	homeobox A13	0.37821497473876103	0.0	0.3472222222222222	0.0	0.0	0.04582283617907244	0.10360530509767855	0.0
FUBP1	far upstream element binding protein 1	0.3781801528985051	0.0	0.3654835390946502	0.0	0.0	0.0	0.0507864552154195	0.0
EZH2	enhancer of zeste 2 polycomb repressive complex 2 subunit	0.3768659604906673	0.0	0.2777777777777778	0.0	0.0	0.009991746236453524	0.30631132201995026	0.0
CSMD3	CUB and Sushi multiple domains 3	0.37167700617283955	0.0	0.36627700617283954	0.0	0.0	0.0	0.0216	0.0
AFF1	AF4/FMR2 family member 1	0.36691141975308633	0.0	0.35621141975308634	0.0	0.0	0.0	0.042800000000000005	0.0
EPHA3	EPH receptor A3	0.36475591159030224	0.0	0.3095619658119658	0.2	0.0	0.005014852800555992	0.04392465730471516	0.0
DAXX	death domain associated protein	0.36323055555555556	0.0	0.3472222222222222	0.0	0.0	0.0	0.06403333333333333	0.0
EPHA7	EPH receptor A7	0.3596064814814815	0.0	0.3096064814814815	0.2	0.0	0.0	0.0	0.0
PHF6	PHD finger protein 6	0.3578222222222222	0.0	0.3472222222222222	0.0	0.0	0.0	0.0424	0.0
NSD1	nuclear receptor binding SET domain protein 1	0.3563331433998101	0.0	0.3517331433998101	0.0	0.0	0.0	0.0184	0.0
CNBP	CCHC-type zinc finger nucleic acid binding protein	0.35512222222222223	0.0	0.3472222222222222	0.0	0.0	0.0	0.0316	0.0
NPM1	nucleophosmin 1	0.3549764309072029	0.0	0.2777777777777778	0.0	0.0	0.0	0.2855319864627584	0.0
SRC	SRC proto-oncogene, non-receptor tyrosine kinase	0.3545552372118658	0.0	0.2777777777777778	0.2611111111111111	0.0	0.0	0.10349713490679226	0.0
NBEA	neurobeachin	0.35441088078897653	0.0	0.3533599887766554	0.0	0.0	0.004203568049284553	0.0	0.0
KTN1	kinectin 1	0.3543095238095238	0.0	0.3363095238095238	0.0	0.0	0.0	0.07200000000000001	0.0
CDKN2C	cyclin dependent kinase inhibitor 2C	0.353470756638802	0.0	0.3472222222222222	0.0	0.0	0.024994137666319298	0.0	0.0

SMARCE1	SWI/SNF related, matrix associated, actin dependent regulator of chromatin, subfamily e, member 1	0.3534222222222222	0.0	0.3472222222222222	0.0	0.0	0.0	0.024800000000000003	0.0
STAT5B	signal transducer and activator of transcription 5B	0.3524222222222222	0.0	0.3472222222222222	0.0	0.0	0.0	0.020800000000000003	0.0
THRAP3	thyroid hormone receptor associated protein 3	0.3505940329218106	0.0	0.34889403292181065	0.0	0.0	0.0	0.006800000000000005	0.0
TNC	tenascin C	0.3483800095559295	0.0	0.32417052469135804	0.0	0.0	0.0	0.09683793945828595	0.0
ITGAV	integrin subunit alpha V	0.3477904299546446	0.0	0.30989583333333337	0.0	0.0	0.0	0.15157838648524494	0.0
MYD88	MYD88 innate immune signal transduction adaptor	0.3472222222222222	0.0	0.3472222222222222	0.0	0.0	0.0	0.0	0.0
NAB2	NGFI-A binding protein 2	0.3472222222222222	0.0	0.3472222222222222	0.0	0.0	0.0	0.0	0.0
GNA11	G protein subunit alpha 11	0.3472222222222222	0.0	0.3472222222222222	0.0	0.0	0.0	0.0	0.0
PRKCB	protein kinase C beta	0.346381729234362	0.0	0.3101851851851852	0.1	0.0	0.005554839329534596	0.0976442993197279	0.0
ALDH2	aldehyde dehydrogenase 2 family member	0.34607537101411895	0.0	0.2777777777777778	0.0	0.0	0.06596	0.24387481738980898	0.0
COL3A1	collagen type III alpha 1 chain	0.3460277777777777	0.0	0.3402777777777778	0.0	0.0	0.0	0.023000000000000003	0.0
FOXO1	forkhead box O1	0.34489536439158874	0.0	0.2777777777777778	0.0	0.0	0.006524555800383977	0.2655705438772953	0.0
PTPRD	protein tyrosine phosphatase receptor type D	0.34437967592592594	0.0	0.3096064814814815	0.0	0.0	0.0	0.13909277777777776	0.0
CLIP1	CAP-Gly domain containing linker protein 1	0.3442926143941289	0.0	0.3392857142857143	0.0	0.0	0.020027600433658324	0.0	0.0
COL1A1	collagen type I alpha 1 chain	0.3441660943877551	0.0	0.32114197530864197	0.0	0.0	0.0	0.0920964763164525	0.0
ZEB1	zinc finger E-box binding homeobox 1	0.34395507153780047	0.0	0.2777777777777778	0.0	0.0	0.0	0.2647091750400908	0.0
AKAP9	A-kinase anchoring protein 9	0.34296296296296297	0.0	0.33796296296296297	0.0	0.0	0.0	0.020000000000000004	0.0
PDE4DIP	phosphodiesterase 4D interacting protein	0.3418769028696957	0.0	0.3395061728395062	0.0	0.0	0.009482920120758064	0.0	0.0
FAT3	FAT atypical cadherin 3	0.3400997150997151	0.0	0.3400997150997151	0.0	0.0	0.0	0.0	0.0
GPHN	gephyrin	0.33958333333333335	0.0	0.33958333333333335	0.0	0.0	0.0	0.0	0.0
SPECC1	sperm antigen with calponin homology and coiled-coil domains 1	0.33958333333333335	0.0	0.33958333333333335	0.0	0.0	0.0	0.0	0.0
ECT2L	epithelial cell transforming 2 like	0.3395061728395062	0.0	0.3395061728395062	0.0	0.0	0.0	0.0	0.0
KIF5B	kinesin family member 5B	0.33940972222222227	0.0	0.33940972222222227	0.0	0.0	0.0	0.0	0.0
PPFIBP1	PPFIA binding protein 1	0.33888888888888885	0.0	0.33888888888888885	0.0	0.0	0.0	0.0	0.0
CSF3R	colony stimulating factor 3 receptor	0.3375931359292531	0.0	0.2777777777777778	0.225	0.0	0.003419261287802502	0.030164888888888894	0.0
WNK2	WNK lysine deficient protein kinase 2	0.3367130577850998	0.0	0.32143775720164613	0.0	0.0	0.05025675788937017	0.024400000000000005	0.0
BIRC6	baculoviral IAP repeat containing 6	0.3365693268665491	0.0	0.3233943268665491	0.0	0.0	0.0	0.052700000000000004	0.0
FCGR2B	Fc fragment of IgG receptor IIb	0.3353548088738885	0.0	0.2777777777777778	0.0	0.0	0.08947827986499665	0.0	0.19054000000000001
PTK6	protein tyrosine kinase 6	0.3352111111111111	0.0	0.2777777777777778	0.2	0.0	0.0	0.06690000000000002	0.0
ZNRF3	zinc and ring finger 3	0.33465599678701147	0.0	0.32069830246913583	0.0	0.0	0.05280855504928017	0.006800000000000005	0.0
TPR	translocated promoter region, nuclear basket protein	0.33454838664421993	0.0	0.32674838664421996	0.0	0.0	0.0	0.031200000000000006	0.0
ELF3	E74 like ETS transcription factor 3	0.33408958333333333	0.0	0.2777777777777778	0.0	0.0	0.0	0.22524722222222224	0.0
MSI2	musashi RNA binding protein 2	0.33392435710824997	0.0	0.2777777777777778	0.0	0.0	0.008230963974249373	0.22092811111111113	0.0
IGF2BP2	insulin like growth factor 2 mRNA binding protein 2	0.333539384582392	0.0	0.3113425925925926	0.0	0.0	0.0	0.0887871679591976	0.0

PRDM2	PR/SET domain 2	0.3314768703703704	0.0	0.3096064814814815	0.0	0.0	0.0	0.08748155555555556	0.0
TENT5C	terminal nucleotidyltransferase 5C	0.3311527777777778	0.0	0.2777777777777778	0.0	0.0	0.0	0.21350000000000002	0.0
HSP90AA1	heat shock protein 90 alpha family class A member 1	0.331066112244898	0.0	0.2777777777777778	0.1	0.0	0.0	0.1687088934240363	0.0
RALGDS	ral guanine nucleotide dissociation stimulator	0.3300638888888889	0.0	0.3107638888888889	0.0	0.0	0.0	0.0772	0.0
HSP90AB1	heat shock protein 90 alpha family class B member 1	0.32779023068907887	0.0	0.1388888888888889	0.1	0.0	0.004130470581479387	0.28169874294440306	0.0
SETD1B	SET domain containing 1B, histone lysine methyltransferase	0.32708518518518515	0.0	0.3101851851851852	0.0	0.0	0.0	0.0676	0.0
MUC4	mucin 4, cell surface associated	0.32647872218075774	0.0	0.30927579365079366	0.0	0.0	0.0	0.06881171411985633	0.0
CHD2	chromodomain helicase DNA binding protein 2	0.3254127777777778	0.0	0.2777777777777778	0.0	0.0	0.0	0.0	0.19054000000000001
RNF213	ring finger protein 213	0.32475323387153404	0.0	0.3234739368998628	0.0	0.0	0.005117187886684939	0.0	0.0
MNX1	motor neuron and pancreas homeobox 1	0.3245425925925926	0.0	0.3113425925925926	0.0	0.0	0.0	0.05280000000000001	0.0
CTNND2	catenin delta 2	0.32402044836275345	0.0	0.3096064814814815	0.0	0.0	0.05765586752508776	0.0	0.0
MLLT1	MLLT1 super elongation complex subunit	0.3239638888888889	0.0	0.3107638888888889	0.0	0.0	0.0	0.05280000000000001	0.0
ZMYM3	zinc finger MYM-type containing 3	0.3234310699588477	0.0	0.3234310699588477	0.0	0.0	0.0	0.0	0.0
ZMYM2	zinc finger MYM-type containing 2	0.32242612941288984	0.0	0.3107638888888889	0.0	0.0	0.007735164716008403	0.04321111111111112	0.0
SFPQ	splicing factor proline and glutamine rich	0.3218777777777778	0.0	0.2777777777777778	0.0	0.0	0.0	0.1764	0.0
TNFRSF14	TNF receptor superfamily member 14	0.3216319166666667	0.0	0.2777777777777778	0.0	0.0	0.0	0.17541655555555555	0.0
ZNF429	zinc finger protein 429	0.32094478737997256	0.0	0.32094478737997256	0.0	0.0	0.0	0.0	0.0
ARHGEF10	Rho guanine nucleotide exchange factor 10	0.32069830246913583	0.0	0.32069830246913583	0.0	0.0	0.0	0.0	0.0
PMS1	PMS1 homolog 1, mismatch repair system component	0.32069830246913583	0.0	0.32069830246913583	0.0	0.0	0.0	0.0	0.0
EZR	ezrin	0.32008202778136136	0.0	0.2777777777777778	0.0	0.0	0.003456545950256291	0.1676807573697758	0.0
TRIM31	tripartite motif containing 31	0.31998212272582893	0.30257131465644577	0.0	0.0	0.0	0.004822272624448343	0.0675	0.0
FAM135B	family with sequence similarity 135 member B	0.3199588477366255	0.0	0.3199588477366255	0.0	0.0	0.0	0.0	0.0
A1CF	APOBEC1 complementation factor	0.31991419444444447	0.0	0.3107638888888889	0.0	0.0	0.0	0.03660122222222222	0.0
SEPTIN9	septin 9	0.3178138888888889	0.0	0.3107638888888889	0.0	0.0	0.0	0.02820000000000003	0.0
NONO	non-POU domain containing octamer binding	0.3175175925925926	0.0	0.3113425925925926	0.0	0.0	0.0	0.0247	0.0
MAPK1	mitogen-activated protein kinase 1	0.3159232570820043	0.0	0.2777777777777778	0.0	0.0	0.0	0.15258191721690614	0.0
ARHGEF10L	Rho guanine nucleotide exchange factor 10 like	0.3158198412698413	0.0	0.3100198412698413	0.0	0.0	0.0	0.0232	0.0
GLI1	GLI family zinc finger 1	0.3150847447412313	0.0	0.2777777777777778	0.0	0.0	0.0	0.14922786785381423	0.0
BMP5	bone morphogenetic protein 5	0.3150477180213157	0.0	0.2777777777777778	0.0	0.0	0.14907976097415157	0.0	0.0
ELN	elastin	0.3147002811979554	0.0	0.2777777777777778	0.0	0.0	0.019789584922263306	0.13889464260414908	0.0
FBLN2	fibulin 2	0.31464799290669454	0.0	0.3104166666666667	0.0	0.0	0.016925304960111465	0.0	0.0
KLK2	kallikrein related peptidase 2	0.3134425925925926	0.0	0.3113425925925926	0.0	0.0	0.0	0.00840000000000001	0.0
TRIP11	thyroid hormone receptor interactor 11	0.3131537313059805	0.0	0.3101851851851852	0.0	0.0	0.011874184483181251	0.0	0.0

HEY1	hes related family bHLH transcription factor with YRPW motif 1	0.31260441666666666	0.0	0.2777777777777778	0.0	0.0	0.0	0.13930655555555557	0.0
EIF4A2	eukaryotic translation initiation factor 4A2	0.3125	0.0	0.3125	0.0	0.0	0.0	0.0	0.0
CANT1	calcium activated nucleotidase 1	0.31153052736622083	0.0	0.3101851851851852	0.0	0.0	0.0053813687241426175	0.0	0.0
MB21D2	Mab-21 domain containing 2	0.3113425925925926	0.0	0.3113425925925926	0.0	0.0	0.0	0.0	0.0
NUMA1	nuclear mitotic apparatus protein 1	0.3113425925925926	0.0	0.3113425925925926	0.0	0.0	0.0	0.0	0.0
USP44	ubiquitin specific peptidase 44	0.3113425925925926	0.0	0.3113425925925926	0.0	0.0	0.0	0.0	0.0
EML4	EMAP like 4	0.3107638888888889	0.0	0.3107638888888889	0.0	0.0	0.0	0.0	0.0
TCF12	transcription factor 12	0.3107638888888889	0.0	0.3107638888888889	0.0	0.0	0.0	0.0	0.0
GOLGA5	golgin A5	0.3107638888888889	0.0	0.3107638888888889	0.0	0.0	0.0	0.0	0.0
FOXO3	forkhead box O3	0.3104980510991434	0.0	0.2777777777777778	0.0	0.0	0.0	0.13088109328546238	0.0
DCTN1	dynactin subunit 1	0.3104166666666667	0.0	0.3104166666666667	0.0	0.0	0.0	0.0	0.0
DCAF12L2	DDB1 and CUL4 associated factor 12 like 2	0.3104166666666667	0.0	0.3104166666666667	0.0	0.0	0.0	0.0	0.0
ARAF	A-Raf proto-oncogene, serine/threonine kinase	0.3104166666666667	0.0	0.3104166666666667	0.0	0.0	0.0	0.0	0.0
GAS7	growth arrest specific 7	0.3104166666666667	0.0	0.3104166666666667	0.0	0.0	0.0	0.0	0.0
FGFR1OP	FGFR1 oncogene partner	0.3101851851851852	0.0	0.3101851851851852	0.0	0.0	0.0	0.0	0.0
RGS7	regulator of G protein signaling 7	0.3101851851851852	0.0	0.3101851851851852	0.0	0.0	0.0	0.0	0.0
NIN	ninein	0.3100198412698413	0.0	0.3100198412698413	0.0	0.0	0.0	0.0	0.0
NUP214	nucleoporin 214	0.3097993827160494	0.0	0.3097993827160494	0.0	0.0	0.0	0.0	0.0
GRM3	glutamate metabotropic receptor 3	0.3096064814814815	0.0	0.3096064814814815	0.0	0.0	0.0	0.0	0.0
CNTNAP2	contactin associated protein like 2	0.3096064814814815	0.0	0.3096064814814815	0.0	0.0	0.0	0.0	0.0
SET	SET nuclear proto-oncogene	0.3084777777777778	0.0	0.2777777777777778	0.0	0.0	0.0	0.1228	0.0
CNOT1	CCR4-NOT transcription complex subunit 1	0.3068736384145513	0.0	0.1388888888888889	0.0	0.2721514161923291	0.0	0.0	0.0
ITK	IL2 inducible T cell kinase	0.30528888888888894	0.0	0.2777777777777778	0.1	0.0	0.0	0.022600000000000006	0.0
MSN	moesin	0.3036215277777778	0.0	0.2777777777777778	0.0	0.0	0.0	0.10337500000000001	0.0
BTG1	BTG anti-proliferation factor 1	0.3010273888888889	0.0	0.2777777777777778	0.0	0.0	0.0	0.09299844444444444	0.0
CCNE1	cyclin E1	0.3009519296825389	0.0	0.2777777777777778	0.0	0.0	0.07252	0.04539736714285023	0.0
JUN	Jun proto-oncogene, AP-1 transcription factor subunit	0.30016624117909696	0.0	0.2777777777777778	0.0	0.0	0.04656251592927088	0.06885940208115644	0.0
HOXA9	homeobox A9	0.30014416666666666	0.0	0.2777777777777778	0.0	0.0	0.0	0.08946555555555558	0.0
CCR7	C-C motif chemokine receptor 7	0.30009638104266984	0.0	0.2777777777777778	0.0	0.0	0.009956824866057742	0.08484915756354251	0.0
SGK1	serum/glucocorticoid regulated kinase 1	0.2993577991303498	0.0	0.2777777777777778	0.0	0.0	0.0076491921731484365	0.08292044444444445	0.0
CDX2	caudal type homeobox 2	0.2990950366591081	0.0	0.2777777777777778	0.0	0.0	0.0	0.08526903552532122	0.0
PRRX1	paired related homeobox 1	0.2988570277777778	0.0	0.2777777777777778	0.0	0.0	0.0	0.08431699999999999	0.0
WWTR1	WW domain containing transcription regulator 1	0.2985555555555556	0.0	0.2777777777777778	0.0	0.0	0.0	0.0831111111111111	0.0
B2M	beta-2-microglobulin	0.29711809625039715	0.0	0.2777777777777778	0.0	0.0	0.0	0.07736127389047734	0.0
TSHR	thyroid stimulating hormone receptor	0.2967521801050032	0.0	0.2777777777777778	0.0	0.0	0.009669620945028422	0.07160000000000001	0.0
MDM4	MDM4 regulator of p53	0.2961560179988662	0.0	0.2777777777777778	0.0	0.0	0.0	0.07351296088435375	0.0

PDGFB	platelet derived growth factor subunit B	0.2958027777777778	0.0	0.2777777777777778	0.0	0.0	0.0	0.0721	0.0
FLNA	filamin A	0.2953801111111111	0.0	0.2777777777777778	0.0	0.0	0.0	0.07040933333333335	0.0
PABPC1	poly(A) binding protein cytoplasmic 1	0.29381263038548755	0.0	0.2777777777777778	0.0	0.0	0.0	0.06413941043083901	0.0
BCL10	BCL10 immune signaling adaptor	0.2931444444444443	0.0	0.2777777777777778	0.0	0.0	0.0	0.06146666666666666	0.0
IL21R	interleukin 21 receptor	0.2930777777777777	0.0	0.2777777777777778	0.0	0.0	0.0	0.06120000000000004	0.0
HOXD13	homeobox D13	0.292862427512434	0.0	0.2777777777777778	0.0	0.0	0.06033859893862489	0.0	0.0
QKI	QKI, KH domain containing RNA binding	0.2924725421120653	0.0	0.2777777777777778	0.0	0.0	0.0038028790085876314	0.0570888888888889	0.0
GMPS	guanine monophosphate synthase	0.2924422983563337	0.0	0.2777777777777778	0.0	0.0	0.010393103574349988	0.05403892517006803	0.0
PRKAR1A	protein kinase cAMP-dependent type I regulatory subunit alpha	0.29204539909297056	0.0	0.2777777777777778	0.0	0.0	0.0	0.05707048526077098	0.0
PCBP1	poly(rC) binding protein 1	0.29165	0.0	0.2777777777777778	0.0	0.0	0.0	0.05548888888888886	0.0
RSPO2	R-spondin 2	0.2908777777777778	0.0	0.2777777777777778	0.0	0.0	0.0	0.0524	0.0
CCND2	cyclin D2	0.290192531611336	0.0	0.2777777777777778	0.0	0.0	0.0	0.04965901533423286	0.0
PAX8	paired box 8	0.29009175000000004	0.0	0.2777777777777778	0.0	0.0	0.0	0.0492558888888889	0.0
POLG	DNA polymerase gamma, catalytic subunit	0.2899777777777778	0.0	0.2777777777777778	0.0	0.0	0.0	0.0488	0.0
GPC5	glypican 5	0.2898777777777778	0.0	0.2777777777777778	0.0	0.0	0.0	0.0484	0.0
RNASE1	ribonuclease A family member 1, pancreatic	0.2897160555555556	0.0	0.2777777777777778	0.0	0.0	0.0	0.047753111111111114	0.0
PMEPA1	prostate transmembrane protein, androgen induced 1	0.28966181669903085	0.0	0.2777777777777778	0.0	0.0	0.0034563502912772024	0.04600000000000006	0.0
GATA1	GATA binding protein 1	0.2890027777777778	0.0	0.2777777777777778	0.0	0.0	0.0	0.04489999999999995	0.0
CIITA	class II major histocompatibility complex transactivator	0.2887944444444446	0.0	0.2777777777777778	0.0	0.0	0.0	0.04406666666666667	0.0
ETV5	ETS variant 5	0.2887277777777778	0.0	0.2777777777777778	0.0	0.0	0.0	0.04380000000000006	0.0
NFIB	nuclear factor I B	0.2882777777777778	0.0	0.2777777777777778	0.0	0.0	0.0	0.042	0.0
LCP1	lymphocyte cytosolic protein 1	0.2879027777777778	0.0	0.2777777777777778	0.0	0.0	0.0	0.0405	0.0
FANCF	FA complementation group F	0.28782916285152715	0.0	0.2777777777777778	0.0	0.0	0.01216246566374419	0.0348	0.0
ROBO2	roundabout guidance receptor 2	0.28742569903743476	0.0	0.2777777777777778	0.0	0.0	0.021356291336912754	0.02910000000000004	0.0
TCEA1	transcription elongation factor A1	0.2869777777777778	0.0	0.2777777777777778	0.0	0.0	0.0	0.0368	0.0
MPL	MPL proto-oncogene, thrombopoietin receptor	0.2864523333333336	0.0	0.2777777777777778	0.0	0.0	0.0	0.03469822222222222	0.0
EED	embryonic ectoderm development	0.2862444444444447	0.0	0.2777777777777778	0.0	0.0	0.0	0.03386666666666667	0.0
RHOA	ras homolog family member A	0.2858305555555556	0.0	0.2777777777777778	0.0	0.0	0.0	0.032211111111111114	0.0
GATA3	GATA binding protein 3	0.2857222222222222	0.0	0.2777777777777778	0.0	0.0	0.0	0.03177777777777778	0.0
CCNB1IP1	cyclin B1 interacting protein 1	0.2838638888888889	0.0	0.2777777777777778	0.0	0.0	0.0	0.024344444444444445	0.0
TFEB	transcription factor EB	0.2834527777777778	0.0	0.2777777777777778	0.0	0.0	0.0	0.0227	0.0
CREB3L2	cAMP responsive element binding protein 3 like 2	0.2827777777777778	0.0	0.2777777777777778	0.0	0.0	0.0	0.02000000000000004	0.0
KIAA1549	KIAA1549	0.2827777777777778	0.0	0.2777777777777778	0.0	0.0	0.0	0.02000000000000004	0.0
FOXL2	forkhead box L2	0.2823777777777778	0.0	0.2777777777777778	0.0	0.0	0.0	0.0184	0.0

DGCR8	DGCR8 microprocessor complex subunit	0.2820138888888889	0.0	0.2777777777777778	0.0	0.0	0.0	0.01694444444444445	0.0
ASPSCR1	ASPSCR1 tether for SLC2A4, UBX domain containing	0.2818777777777778	0.0	0.2777777777777778	0.0	0.0	0.0	0.016399999999999998	0.0
H3F3B	H3 histone family member 3B	0.2816501640261795	0.0	0.2777777777777778	0.0	0.0	0.015489544993606847	0.0	0.0
SIX2	SIX homeobox 2	0.2814777777777777	0.0	0.2777777777777778	0.0	0.0	0.0	0.014800000000000002	0.0
RAD17	RAD17 checkpoint clamp loader component	0.2813777777777778	0.0	0.2777777777777778	0.0	0.0	0.0	0.014400000000000003	0.0
LONP1	lon peptidase 1, mitochondrial	0.28095192754064446	0.0	0.2777777777777778	0.0	0.0	0.012696599051466707	0.0	0.0
CRLF2	cytokine receptor like factor 2	0.280754841900166	0.0	0.2777777777777778	0.0	0.0	0.01190825648955271	0.0	0.0
NCOA1	nuclear receptor coactivator 1	0.2803971798783511	0.0	0.1736111111111111	0.0	0.0	0.010434001558221405	0.2358350685941043	0.0
DDB2	damage specific DNA binding protein 2	0.2798527777777778	0.0	0.2777777777777778	0.0	0.0	0.0	0.0083	0.0
TRIM27	tripartite motif containing 27	0.2797777777777778	0.0	0.2777777777777778	0.0	0.0	0.0	0.008	0.0
PSIP1	PC4 and SFRS1 interacting protein 1	0.27949671189474173	0.0	0.2777777777777778	0.0	0.0	0.006875736467855812	0.0	0.0
CRTC3	CREB regulated transcription coactivator 3	0.2794777777777777	0.0	0.2777777777777778	0.0	0.0	0.0	0.00680000000000000005	0.0
FES	FES proto-oncogene, tyrosine kinase	0.27886497104058033	0.0	0.2777777777777778	0.0	0.0	0.004348773051210141	0.0	0.0
LSM14A	LSM14A mRNA processing body assembly factor	0.2777777777777778	0.0	0.2777777777777778	0.0	0.0	0.0	0.0	0.0
BAZ1A	bromodomain adjacent to zinc finger domain 1A	0.2777777777777778	0.0	0.2777777777777778	0.0	0.0	0.0	0.0	0.0
FAM47C	family with sequence similarity 47 member C	0.2777777777777778	0.0	0.2777777777777778	0.0	0.0	0.0	0.0	0.0
MYO5A	myosin VA	0.2777777777777778	0.0	0.2777777777777778	0.0	0.0	0.0	0.0	0.0
NACA	nascent polypeptide associated complex subunit alpha	0.2777777777777778	0.0	0.2777777777777778	0.0	0.0	0.0	0.0	0.0
SRGAP3	SLIT-ROBO Rho GTPase activating protein 3	0.2777777777777778	0.0	0.2777777777777778	0.0	0.0	0.0	0.0	0.0
FNBP1	formin binding protein 1	0.2777777777777778	0.0	0.2777777777777778	0.0	0.0	0.0	0.0	0.0
TAL2	TAL bHLH transcription factor 2	0.2777777777777778	0.0	0.2777777777777778	0.0	0.0	0.0	0.0	0.0
ZNF479	zinc finger protein 479	0.2777777777777778	0.0	0.2777777777777778	0.0	0.0	0.0	0.0	0.0
CNBD1	cyclic nucleotide binding domain containing 1	0.2777777777777778	0.0	0.2777777777777778	0.0	0.0	0.0	0.0	0.0
FOXR1	forkhead box R1	0.2777777777777778	0.0	0.2777777777777778	0.0	0.0	0.0	0.0	0.0
CLP1	cleavage and polyadenylation factor I subunit 1	0.2777777777777778	0.0	0.2777777777777778	0.0	0.0	0.0	0.0	0.0
MLLT3	MLLT3 super elongation complex subunit	0.2777777777777778	0.0	0.2777777777777778	0.0	0.0	0.0	0.0	0.0
PWWP2A	PWWP domain containing 2A	0.2777777777777778	0.0	0.2777777777777778	0.0	0.0	0.0	0.0	0.0
TPM4	tropomyosin 4	0.2777777777777778	0.0	0.2777777777777778	0.0	0.0	0.0	0.0	0.0
ACSL6	acyl-CoA synthetase long chain family member 6	0.2777777777777778	0.0	0.2777777777777778	0.0	0.0	0.0	0.0	0.0
FCRL4	Fc receptor like 4	0.2777777777777778	0.0	0.2777777777777778	0.0	0.0	0.0	0.0	0.0
RBM15	RNA binding motif protein 15	0.2777777777777778	0.0	0.2777777777777778	0.0	0.0	0.0	0.0	0.0
U2AF1	U2 small nuclear RNA auxiliary factor 1	0.2777777777777778	0.0	0.2777777777777778	0.0	0.0	0.0	0.0	0.0
FAM131B	family with sequence similarity 131 member B	0.2777777777777778	0.0	0.2777777777777778	0.0	0.0	0.0	0.0	0.0

SLC45A3	solute carrier family 45 member 3	0.2777777777777778	0.0	0.2777777777777778	0.0	0.0	0.0	0.0	0.0
ARHGAP26	Rho GTPase activating protein 26	0.2777777777777778	0.0	0.2777777777777778	0.0	0.0	0.0	0.0	0.0
FIP1L1	factor interacting with PAPOLA and CPSF1	0.2777777777777778	0.0	0.2777777777777778	0.0	0.0	0.0	0.0	0.0
ASXL2	ASXL transcriptional regulator 2	0.2777777777777778	0.0	0.2777777777777778	0.0	0.0	0.0	0.0	0.0
VAV1	vav guanine nucleotide exchange factor 1	0.2777777777777778	0.0	0.2777777777777778	0.0	0.0	0.0	0.0	0.0
MBD1	methyl-CpG binding domain protein 1	0.2777777777777778	0.0	0.2777777777777778	0.0	0.0	0.0	0.0	0.0
KAT7	lysine acetyltransferase 7	0.2777777777777778	0.0	0.2777777777777778	0.0	0.0	0.0	0.0	0.0
TMEM127	transmembrane protein 127	0.2777777777777778	0.0	0.2777777777777778	0.0	0.0	0.0	0.0	0.0
TEC	tec protein tyrosine kinase	0.2777777777777778	0.0	0.2777777777777778	0.0	0.0	0.0	0.0	0.0
MYH11	myosin heavy chain 11	0.2777777777777778	0.0	0.2777777777777778	0.0	0.0	0.0	0.0	0.0
HOXD11	homeobox D11	0.2777777777777778	0.0	0.2777777777777778	0.0	0.0	0.0	0.0	0.0
SBDS	SBDS ribosome maturation factor	0.2777777777777778	0.0	0.2777777777777778	0.0	0.0	0.0	0.0	0.0
SEPTIN6	septin 6	0.2777777777777778	0.0	0.2777777777777778	0.0	0.0	0.0	0.0	0.0
HOXC11	homeobox C11	0.2777777777777778	0.0	0.2777777777777778	0.0	0.0	0.0	0.0	0.0
CEP89	centrosomal protein 89	0.2777777777777778	0.0	0.2777777777777778	0.0	0.0	0.0	0.0	0.0
KDSR	3-ketodihydrosphingosine reductase	0.2777777777777778	0.0	0.2777777777777778	0.0	0.0	0.0	0.0	0.0
CNTRL	centriolin	0.2777777777777778	0.0	0.2777777777777778	0.0	0.0	0.0	0.0	0.0
STRN	striatin	0.2777777777777778	0.0	0.2777777777777778	0.0	0.0	0.0	0.0	0.0
CNOT6	CCR4-NOT transcription complex subunit 6	0.2777777777777778	0.0	0.2777777777777778	0.0	0.0	0.0	0.0	0.0
POU2AF1	POU class 2 homeobox associating factor 1	0.2777777777777778	0.0	0.2777777777777778	0.0	0.0	0.0	0.0	0.0
YWHAE	tyrosine 3-monooxygenase/tryptophan 5-monooxygenase activation protein epsilon	0.2777777777777778	0.0	0.2777777777777778	0.0	0.0	0.0	0.0	0.0
LARP4B	La ribonucleoprotein domain family member 4B	0.2777777777777778	0.0	0.2777777777777778	0.0	0.0	0.0	0.0	0.0
LYL1	LYL1 basic helix-loop-helix family member	0.2777777777777778	0.0	0.2777777777777778	0.0	0.0	0.0	0.0	0.0
PATZ1	POZ/BTB and AT hook containing zinc finger 1	0.2777777777777778	0.0	0.2777777777777778	0.0	0.0	0.0	0.0	0.0
ERC1	ELKS/RAB6-interacting/CAST family member 1	0.2777777777777778	0.0	0.2777777777777778	0.0	0.0	0.0	0.0	0.0
N4BP2	NEDD4 binding protein 2	0.2777777777777778	0.0	0.2777777777777778	0.0	0.0	0.0	0.0	0.0
NT5C2	5'-nucleotidase, cytosolic II	0.2777777777777778	0.0	0.2777777777777778	0.0	0.0	0.0	0.0	0.0
PICALM	phosphatidylinositol binding clathrin assembly protein	0.2777777777777778	0.0	0.2777777777777778	0.0	0.0	0.0	0.0	0.0
CDH10	cadherin 10	0.2777777777777778	0.0	0.2777777777777778	0.0	0.0	0.0	0.0	0.0
CUL3	cullin 3	0.2777777777777778	0.0	0.2777777777777778	0.0	0.0	0.0	0.0	0.0
ANK1	ankyrin 1	0.2777777777777778	0.0	0.2777777777777778	0.0	0.0	0.0	0.0	0.0
BCLAF1	BCL2 associated transcription factor 1	0.2777777777777778	0.0	0.2777777777777778	0.0	0.0	0.0	0.0	0.0
PAX7	paired box 7	0.2777777777777778	0.0	0.2777777777777778	0.0	0.0	0.0	0.0	0.0
ETV1	ETS variant 1	0.2777777777777778	0.0	0.2777777777777778	0.0	0.0	0.0	0.0	0.0

LASP1	LIM and SH3 protein 1	0.27462496846853274	0.0	0.1388888888888889	0.0	0.0	0.0	0.23990274624631053	0.0
HLA-DQA2	major histocompatibility complex, class II, DQ alpha 2	0.27406162498721187	0.27406162498721187	0.0	0.0	0.0	0.0	0.0	0.0
ISX	intestine specific homeobox	0.27081555555555553	0.0	0.1388888888888889	0.0	0.0	0.12078	0.22267333333333333	0.0
SAMM50	SAMM50 sorting and assembly machinery component	0.26198282459096256	0.26198282459096256	0.0	0.0	0.0	0.0	0.0	0.0
AKT3	AKT serine/threonine kinase 3	0.24503114073654153	0.0	0.1388888888888889	0.2	0.0	0.0	0.09278026662887376	0.0
IL2	interleukin 2	0.2396224876216162	0.0	0.1388888888888889	0.0	0.0	0.0	0.12924238859454548	0.19054000000000001
CCR4	C-C motif chemokine receptor 4	0.23575221838122412	0.047919965431016995	0.0	0.1	0.0	0.0	0.2054277777777778	0.0
ATIC	5-aminoimidazole-4-carboxamide ribonucleotide formyltransferase/IMP cyclohydrolase	0.2347222222222222	0.0	0.1388888888888889	0.0	0.0	0.0	0.2	0.0
SMC1A	structural maintenance of chromosomes 1A	0.2347222222222222	0.0	0.1388888888888889	0.0	0.0	0.0	0.2	0.0
CYP26B1	cytochrome P450 family 26 subfamily B member 1	0.21003433318879938	0.21003433318879938	0.0	0.0	0.0	0.0	0.0	0.0
CPEB3	cytoplasmic polyadenylation element binding protein 3	0.20064196994721709	0.0	0.1388888888888889	0.0	0.0	0.01067772952495369	0.16473333333333334	0.0
TFRC	transferrin receptor	0.19168514882581333	0.0	0.1388888888888889	0.0	0.0	0.0	0.15696292660359112	0.0
CDH17	cadherin 17	0.1863889917357961	0.0	0.1388888888888889	0.0	0.0	0.0	0.15166676951357388	0.0
CHST11	carbohydrate sulfotransferase 11	0.1859111111111111	0.0	0.1736111111111111	0.0	0.0	0.0	0.04920000000000001	0.0
PTPN6	protein tyrosine phosphatase non-receptor type 6	0.18427116666666665	0.0	0.1736111111111111	0.0	0.0	0.0	0.042640222222222225	0.0
CRNKL1	crooked neck pre-mRNA splicing factor 1	0.17729300276108187	0.0	0.1736111111111111	0.0	0.0	0.014727566599883066	0.0	0.0
TMPRSS2	transmembrane serine protease 2	0.1736111111111111	0.0	0.1736111111111111	0.0	0.0	0.0	0.0	0.0
BPTF	bromodomain PHD finger transcription factor	0.1616465277777778	0.0	0.1388888888888889	0.0	0.0	0.0	0.09103055555555557	0.0
CASP9	caspase 9	0.15989100590150712	0.0	0.1388888888888889	0.0	0.0	0.0	0.08400846805047293	0.0
BCR	BCR activator of RhoGEF and GTPase	0.15634947019905568	0.0	0.1388888888888889	0.0	0.0	0.0032452317915009628	0.0684	0.0
SOX21	SRY-box 21	0.1527888888888889	0.0	0.1388888888888889	0.0	0.0	0.0	0.05560000000000001	0.0
BCL2L12	BCL2 like 12	0.1522888888888889	0.0	0.1388888888888889	0.0	0.0	0.0	0.05360000000000001	0.0
FEN1	flap structure-specific endonuclease 1	0.1511035004286339	0.0	0.1388888888888889	0.0	0.0	0.025556503857705064	0.0375	0.0
RTN4	reticulon 4	0.15094244444444443	0.0	0.1388888888888889	0.0	0.0	0.0	0.048214222222222214	0.0
JAZF1	JAZF zinc finger 1	0.15030555555555555	0.0	0.1388888888888889	0.0	0.0	0.0	0.045666666666666675	0.0
CCNC	cyclin C	0.1501888888888889	0.0	0.1388888888888889	0.0	0.0	0.0	0.045200000000000004	0.0
CD209	CD209 molecule	0.1488888888888889	0.0	0.1388888888888889	0.0	0.0	0.0	0.04000000000000001	0.0
PRCC	proline rich mitotic checkpoint control factor	0.1463888888888889	0.0	0.1388888888888889	0.0	0.0	0.0	0.03	0.0
NFATC4	nuclear factor of activated T cells 4	0.1463888888888889	0.0	0.1388888888888889	0.0	0.0	0.0	0.03	0.0
ID3	inhibitor of DNA binding 3, HLH protein	0.1458888888888889	0.0	0.1388888888888889	0.0	0.0	0.0	0.028000000000000004	0.0
ARHGAP35	Rho GTPase activating protein 35	0.1453388888888889	0.0	0.1388888888888889	0.0	0.0	0.0	0.025800000000000003	0.0
NCOA4	nuclear receptor coactivator 4	0.1425888888888889	0.0	0.1388888888888889	0.0	0.0	0.0	0.014800000000000002	0.0

SS18L1	SS18L1 subunit of BAF chromatin remodeling complex	0.1418888888888889	0.0	0.1388888888888889	0.0	0.0	0.0	0.012	0.0
KNSTRN	kinetochore localized astrin (SPAG5) binding protein	0.14002497490536578	0.0	0.1388888888888889	0.0	0.0	0.004544344065907486	0.0	0.0
SHTN1	shootin 1	0.13976944257534427	0.0	0.1388888888888889	0.0	0.0	0.0035222147458215305	0.0	0.0
AC000093.1	novel transcript	0.1388888888888889	0.0	0.1388888888888889	0.0	0.0	0.0	0.0	0.0
HIST1H4I	histone cluster 1 H4 family member i	0.1388888888888889	0.0	0.1388888888888889	0.0	0.0	0.0	0.0	0.0
MALAT1	metastasis associated lung adenocarcinoma transcript 1	0.1388888888888889	0.0	0.1388888888888889	0.0	0.0	0.0	0.0	0.0
ITSN1	intersectin 1	0.1388888888888889	0.0	0.1388888888888889	0.0	0.0	0.0	0.0	0.0
CEP290	centrosomal protein 290	0.1388888888888889	0.0	0.1388888888888889	0.0	0.0	0.0	0.0	0.0
MDS2	myelodysplastic syndrome 2 translocation associated	0.1388888888888889	0.0	0.1388888888888889	0.0	0.0	0.0	0.0	0.0
NUTM2A	NUT family member 2A	0.1388888888888889	0.0	0.1388888888888889	0.0	0.0	0.0	0.0	0.0
HOOK3	hook microtubule tethering protein 3	0.1388888888888889	0.0	0.1388888888888889	0.0	0.0	0.0	0.0	0.0
COX6C	cytochrome c oxidase subunit 6C	0.1388888888888889	0.0	0.1388888888888889	0.0	0.0	0.0	0.0	0.0
WDCP	WD repeat and coiled coil containing	0.1388888888888889	0.0	0.1388888888888889	0.0	0.0	0.0	0.0	0.0
LMNA	lamin A/C	0.1388888888888889	0.0	0.1388888888888889	0.0	0.0	0.0	0.0	0.0
CYSLTR2	cysteinyl leukotriene receptor 2	0.1388888888888889	0.0	0.1388888888888889	0.0	0.0	0.0	0.0	0.0
S100A7	S100 calcium binding protein A7	0.1388888888888889	0.0	0.1388888888888889	0.0	0.0	0.0	0.0	0.0
SS18	SS18 subunit of BAF chromatin remodeling complex	0.1388888888888889	0.0	0.1388888888888889	0.0	0.0	0.0	0.0	0.0
MYH10	myosin heavy chain 10	0.1388888888888889	0.0	0.1388888888888889	0.0	0.0	0.0	0.0	0.0
MACF1	microtubule actin crosslinking factor 1	0.1388888888888889	0.0	0.1388888888888889	0.0	0.0	0.0	0.0	0.0
FKBP1B	FKBP prolyl isomerase 1B	0.1388888888888889	0.0	0.1388888888888889	0.0	0.0	0.0	0.0	0.0
ASH1L	ASH1 like histone lysine methyltransferase	0.1388888888888889	0.0	0.1388888888888889	0.0	0.0	0.0	0.0	0.0
BCL7A	BAF chromatin remodeling complex subunit BCL7A	0.1388888888888889	0.0	0.1388888888888889	0.0	0.0	0.0	0.0	0.0
TFPT	TCF3 fusion partner	0.1388888888888889	0.0	0.1388888888888889	0.0	0.0	0.0	0.0	0.0
LEPROTL1	leptin receptor overlapping transcript like 1	0.1388888888888889	0.0	0.1388888888888889	0.0	0.0	0.0	0.0	0.0
HHAT	hedgehog acyltransferase	0.1388888888888889	0.0	0.1388888888888889	0.0	0.0	0.0	0.0	0.0
HERPUD1	homocysteine inducible ER protein with ubiquitin like domain 1	0.1388888888888889	0.0	0.1388888888888889	0.0	0.0	0.0	0.0	0.0
RABEP1	rabaptin, RAB GTPase binding effector protein 1	0.1388888888888889	0.0	0.1388888888888889	0.0	0.0	0.0	0.0	0.0
TLL1	tolloid like 1	0.08057964666886383	0.051963031119899715	0.0	0.0	0.0	0.0	0.0675888888888889	0.0
EFCAB11	EF-hand calcium binding domain 11	0.05686289581001867	0.050197243453977504	0.0	0.0	0.0	0.011390871204370464	0.021600000000000005	0.0
C2	complement C2	0.052996621726988755	0.052996621726988755	0.0	0.0	0.0	0.0	0.0	0.0
VEPH1	ventricular zone expressed PH	0.03259810842480203	0.03259810842480203	0.0	0.0	0.0	0.0	0.0	0.0

	domain containing 1								
DLC1	DLC1 Rho GTPase activating protein	0.0219289472 9757386	0.0157157891 90295426	0.0	0.0	0.0	0.0	0.01800000000 0000002	0.0
AC0134 89.1	novel transcript	0.0124198771 22258983	0.0124198771 22258983	0.0	0.0	0.0	0.0	0.0	0.0
HS3ST1	heparan sulfate-glucosamine 3-sulfotransferase 1	0.0104084574 10619371	0.0095537516 3250691	0.0	0.0	0.0	0.00341882311 24498434	0.0	0.0
AGA	aspartylglucosa minidase	0.0096778263 49288978	0.0096778263 49288978	0.0	0.0	0.0	0.0	0.0	0.0

Supplementary Table S2. All targets and their genetic associations and somatic mutations scores.

Target gene	Target ID	Score.overall	Genetic association score	Somatic mutation score	Arithmetic mean
TP53	ENSG00000141510	1.0	1.0	1.0	1
CTNNB1	ENSG00000168036	1.0	1.0	1.0	1
TERT	ENSG00000164362	1.0	0.8419444444444443	0.711398898898899	0,776671672
PIK3CA	ENSG00000121879	1.0	0.5	1.0	0,75
AXIN1	ENSG00000103126	1.0	0.0	1.0	0,5
MET	ENSG00000105976	1.0	0.0	0.9915123456790124	0,495756173
NFE2L2	ENSG00000116044	0.9861488324613179	0.0	0.9710778486820153	0,485538924
IDH1	ENSG00000138413	0.9757839263791365	0.0	0.9635802469135802	0,481790123
HRAS	ENSG00000174775	0.9655228070697964	0.0	0.947787628425856	0,473893814
CDKN2A	ENSG00000147889	0.9643727643583659	0.0	0.9375694656423823	0,468784733
CREBBP	ENSG00000005339	0.9353246276167473	0.0	0.9284357387278583	0,464217869
NRAS	ENSG00000213281	0.9276289828184364	0.0	0.917283950617284	0,458641975
IDH2	ENSG00000182054	0.9276793075494287	0.0	0.911033950617284	0,455516975
ARID2	ENSG00000189079	0.8975792476262491	0.0	0.8680265222849968	0,434013261
GNAS	ENSG00000087460	0.8643253914380926	0.0	0.8603395061728395	0,430169753
KRAS	ENSG00000133703	0.8270290899955751	0.0	0.8159722222222222	0,407986111
FGFR1	ENSG00000077782	1.0	0.0	0.798611111111111	0,399305556
TSC2	ENSG00000103197	0.8223728912477103	0.0	0.7777111760883691	0,388855588
SF3B1	ENSG00000115524	0.787411111111111	0.0	0.7638888888888888	0,381944444
APC	ENSG00000134982	0.8617515044760831	0.0	0.744624732905983	0,372312366
HLA-DQA1	ENSG00000196735	0.7225721750469736	0.7225721750469736	0.0	0,361286088
IL6ST	ENSG00000134352	0.7595346974403909	0.0	0.7105475040257648	0,355273752
ARID1A	ENSG00000117713	0.7282025652730801	0.0	0.702991252261052	0,351495626
IGF2R	ENSG00000197081	0.7190700746893237	0.0	0.6944444444444444	0,347222222
RXRA	ENSG00000186350	0.7071105414537384	0.0	0.6944444444444444	0,347222222
CNOT9	ENSG00000144580	0.6957521220793593	0.0	0.6944444444444444	0,347222222
SF3B2	ENSG00000087365	0.6793944444444445	0.0	0.6770833333333334	0,338541667
CASP8	ENSG00000064012	0.6951975105906835	0.0	0.6756365740740741	0,337818287
ACVR2A	ENSG00000121989	0.6696192495126705	0.0	0.6684636939571149	0,334231847
NF1	ENSG00000196712	0.6730377888636936	0.0	0.6680555555555555	0,334027778
KMT2C	ENSG00000055609	0.6657109658678286	0.0	0.6634331880900508	0,331716594
PTEN	ENSG00000171862	0.7142386544095812	0.0	0.661028972520908	0,330514486
ATRX	ENSG00000085224	0.6556311728395061	0.0	0.6556311728395061	0,327815586
KIF1B	ENSG00000054523	0.6995011950776868	0.651450028120844	0.0	0,325725014
PBRM1	ENSG00000163939	0.6632685273368606	0.0	0.6490575396825397	0,32452877
PRKACA	ENSG00000072062	0.625	0.0	0.625	0,3125
HNF1A	ENSG00000135100	0.6552025662516333	0.0	0.5939663973414934	0,296983199
DNAJB1	ENSG00000132002	0.6139797938061801	0.0	0.5902777777777778	0,295138889
TBL1XR1	ENSG00000177565	0.5945910493827161	0.0	0.586111111111111	0,293055556
MAGI1	ENSG00000151276	0.5688180827886711	0.0	0.5688180827886711	0,284409041

ARID1B	ENSG00000049618	0.5795966392318244	0.0	0.5686299725651577	0,284314986
BRCA2	ENSG00000139618	0.5944784103249043	0.0	0.5642010381593715	0,282100519
LRP1B	ENSG00000168702	0.5624908315177922	0.0	0.5609908315177923	0,280495416
TSC1	ENSG00000165699	0.5994610138888888	0.0	0.5599392361111111	0,279969618
ATM	ENSG00000149311	0.662331487312914	0.0	0.5592206790123457	0,27961034
HLA-DQB1	ENSG00000179344	0.5749026631525533	0.558725	0.0	0,2793625
NUP98	ENSG00000110713	0.5586271367521367	0.0	0.5586271367521367	0,279313568
ROS1	ENSG00000047936	0.5609159069586652	0.0	0.5562114197530865	0,27810571
RB1	ENSG00000139687	0.595301117069666	0.0	0.5560875896057348	0,278043795
AKT1	ENSG00000142208	0.6486487590891844	0.0	0.5555555555555556	0,277777778
SETD2	ENSG00000181555	0.6184285314829113	0.0	0.5552083333333334	0,277604167
KDM6A	ENSG00000147050	0.556287682379349	0.0	0.552587682379349	0,276293841
FLT3	ENSG00000122025	1.0	0.0	0.5505952380952381	0,275297619
BLM	ENSG00000197299	0.5740002410698014	0.0	0.5503472222222222	0,275173611
SMARCA4	ENSG00000127616	0.6472277056260018	0.0	0.5491469478737998	0,274573474
NCOR1	ENSG00000141027	0.5533784050785039	0.0	0.5459104938271605	0,272955247
SUZ12	ENSG00000178691	0.5569387304118836	0.0	0.5444315843621399	0,272215792
KMT2A	ENSG00000118058	0.5592055033383725	0.0	0.541550925925926	0,270775463
KMT2D	ENSG00000167548	0.5367155349794239	0.0	0.5367155349794239	0,268357767
AXIN2	ENSG00000168646	0.5695687718501588	0.0	0.5362225651577504	0,268111283
MECOM	ENSG00000085276	0.5502035881634597	0.0	0.5346628679962013	0,267331434
ANTXR1	ENSG00000169604	0.556795869644154	0.0	0.5336555918663761	0,266827796
AMER1	ENSG00000184675	0.5446033950617284	0.0	0.5336033950617284	0,266801698
KEAP1	ENSG00000079999	0.5413374302684604	0.0	0.5334809447128288	0,266740472
FLT4	ENSG00000037280	1.0	0.0	0.5326829805996472	0,26634149
KIT	ENSG00000157404	1.0	0.0	0.5326829805996472	0,26634149
FZR1	ENSG00000105325	0.5324556327160493	0.0	0.5324556327160493	0,266227816
AL031847.2	ENSG00000285629	0.5316358024691358	0.0	0.5316358024691358	0,265817901
TCF7L2	ENSG00000148737	0.7060830726587126	0.0	0.530542695473251	0,265271348
NOTCH1	ENSG00000148400	0.5726441803112589	0.0	0.5268454218106996	0,263422711
FAT4	ENSG00000196159	0.537964420774739	0.0	0.5219650205761316	0,26098251
LATS1	ENSG00000131023	0.5703243854436789	0.0	0.5183917548500881	0,259195877
ASXL1	ENSG00000171456	0.5179694919278253	0.0	0.5179694919278253	0,258984746
ERBB3	ENSG00000065361	0.6022607315794631	0.0	0.5147707231040565	0,257385362
BCOR	ENSG00000183337	0.5244957231040565	0.0	0.5147707231040565	0,257385362
CIC	ENSG00000079432	0.5314521604938272	0.0	0.5146604938271605	0,257330247
TRRAP	ENSG00000196367	0.5783380384583254	0.0	0.5128205128205128	0,256410256
MEN1	ENSG00000133895	0.5328018838708323	0.0	0.5126028806584362	0,25630144
MYH9	ENSG00000100345	0.5126028806584362	0.0	0.5126028806584362	0,25630144
PTPRB	ENSG00000127329	0.5577487863823485	0.0	0.509837962962963	0,254918981
ZFHX3	ENSG00000140836	0.5287114027777777	0.0	0.509765625	0,254882813
NCOR2	ENSG00000196498	0.5097222222222222	0.0	0.5097222222222222	0,254861111
DICER1	ENSG00000100697	0.5239339265285772	0.0	0.509548611111111	0,254774306

COL2A1	ENSG00000139219	0.5122056353070112	0.0	0.509375	0,2546875
MAP3K1	ENSG00000095015	0.5213145833333334	0.0	0.5091145833333334	0,254557292
CACNA1D	ENSG00000157388	0.5091145833333334	0.0	0.5091145833333334	0,254557292
CLTC	ENSG00000141367	0.5151932379349046	0.0	0.5087682379349046	0,254384119
NF2	ENSG00000186575	0.5316305689247922	0.0	0.5086805555555556	0,254340278
TBX3	ENSG00000135111	0.5280305555555556	0.0	0.5086805555555556	0,254340278
KDM5A	ENSG00000073614	0.5199160276441358	0.0	0.5086805555555556	0,254340278
RANBP2	ENSG00000153201	0.5120876543209877	0.0	0.5084876543209876	0,254243827
KDR	ENSG00000128052	1.0	0.0	0.5059156378600823	0,252957819
MSH6	ENSG00000116062	0.5175740249625231	0.0	0.505787037037037	0,252893519
ALK	ENSG00000171094	0.5607288628065256	0.0	0.5055941358024691	0,252797068
JAK3	ENSG00000105639	0.5094803591470257	0.0	0.5049803591470258	0,25249018
SMAD4	ENSG00000141646	0.5669502894919922	0.0	0.5045438957475995	0,252271948
RET	ENSG00000165731	1.0	0.0	0.5042438271604939	0,252121914
PTPRK	ENSG00000152894	0.5096188271604939	0.0	0.5042438271604939	0,252121914
WT1	ENSG00000184937	0.5628203537469423	0.0	0.5038580246913581	0,251929012
ESR1	ENSG00000091831	0.9519781878015823	0.0	0.503343621399177	0,251671811
MYB	ENSG00000118513	0.5201984567901234	0.0	0.5026234567901234	0,251311728
XPO1	ENSG00000082898	0.5811090256000897	0.0	0.5015432098765432	0,250771605
SMAD2	ENSG00000175387	0.5345736926932448	0.0	0.5015432098765432	0,250771605
FLCN	ENSG00000154803	0.5045432098765432	0.0	0.5015432098765432	0,250771605
NTRK1	ENSG00000198400	0.5559287824412941	0.0	0.5	0,25

Supplementary Table S3. Filtration criteria for genes in ChEMBL database.

Criteria	Filtration
Target organism	*Homo sapiens*
Target type	Single protein
Assay type	Binding
Standard unit	Molar
Standard type	IC_{50}
Standard relation	=, <
pChEMBL value	Exist

Supplementary Table S4. Drug target interactions after MDeePred.

COMPOUND ID	TARGET ID
CHEMBL1241270	CHEMBL4005
CHEMBL3763833	CHEMBL2148
CHEMBL1241490	CHEMBL4005
CHEMBL3895671	CHEMBL279
CHEMBL4215501	CHEMBL3717
CHEMBL4112408	CHEMBL4247
CHEMBL2418345	CHEMBL4005
CHEMBL3678511	CHEMBL3717
CHEMBL3671818	CHEMBL2148
CHEMBL4291436	CHEMBL4005
CHEMBL452812	CHEMBL1955
CHEMBL494921	CHEMBL4005
CHEMBL596583	CHEMBL4282
CHEMBL206198	CHEMBL4282
CHEMBL3642255	CHEMBL2148
CHEMBL3648908	CHEMBL4005
CHEMBL3976418	CHEMBL4247
CHEMBL3940191	CHEMBL4247
CHEMBL3915565	CHEMBL2815
CHEMBL570573	CHEMBL279
CHEMBL3319466	CHEMBL1936
CHEMBL2152127	CHEMBL4005
CHEMBL2088106	CHEMBL3650
CHEMBL509435	CHEMBL1974
CHEMBL1760157	CHEMBL4005
CHEMBL3964812	CHEMBL279
CHEMBL2018882	CHEMBL1955
CHEMBL3661583	CHEMBL2815
CHEMBL3948623	CHEMBL279
CHEMBL3134298	CHEMBL4005
CHEMBL1922224	CHEMBL4247
CHEMBL3398170	CHEMBL4247
CHEMBL3286814	CHEMBL4247
CHEMBL230686	CHEMBL279
CHEMBL3753512	CHEMBL2041
CHEMBL4168420	CHEMBL4005
CHEMBL3687221	CHEMBL2041
CHEMBL493083	CHEMBL4005
CHEMBL2402840	CHEMBL3717
CHEMBL1614778	CHEMBL4005
CHEMBL3670213	CHEMBL4005

CHEMBL222539	CHEMBL2916
CHEMBL3753903	CHEMBL2041
CHEMBL1170550	CHEMBL2041
CHEMBL4166436	CHEMBL3717
CHEMBL1098311	CHEMBL4005
CHEMBL2431839	CHEMBL3717
CHEMBL77242	CHEMBL3650
CHEMBL412081	CHEMBL279
CHEMBL3919045	CHEMBL279
CHEMBL352992	CHEMBL279
CHEMBL3967141	CHEMBL279
CHEMBL1940105	CHEMBL1974
CHEMBL196609	CHEMBL279
CHEMBL460989	CHEMBL2815
CHEMBL3754722	CHEMBL279
CHEMBL3401762	CHEMBL3650
CHEMBL246355	CHEMBL279
CHEMBL74143	CHEMBL279
CHEMBL3286808	CHEMBL4247
CHEMBL558306	CHEMBL3717
CHEMBL170984	CHEMBL279
CHEMBL3786830	CHEMBL4247
CHEMBL1242032	CHEMBL279
CHEMBL3644994	CHEMBL2148
CHEMBL493315	CHEMBL279
CHEMBL3286829	CHEMBL4247
CHEMBL2322671	CHEMBL4005
CHEMBL3644956	CHEMBL2148
CHEMBL3667474	CHEMBL2148
CHEMBL191594	CHEMBL279
CHEMBL516429	CHEMBL4282
CHEMBL3775190	CHEMBL279
CHEMBL3695646	CHEMBL4005
CHEMBL185140	CHEMBL2148
CHEMBL3889789	CHEMBL2148
CHEMBL3358977	CHEMBL279
CHEMBL209535	CHEMBL2815
CHEMBL3798482	CHEMBL2148
CHEMBL3612512	CHEMBL4005
CHEMBL572956	CHEMBL4005
CHEMBL3113146	CHEMBL4282
CHEMBL2030439	CHEMBL4005
CHEMBL176705	CHEMBL279

CHEMBL1928728	CHEMBL4005
CHEMBL101797	CHEMBL279
CHEMBL3745943	CHEMBL279
CHEMBL3666286	CHEMBL2815
CHEMBL3951451	CHEMBL279
CHEMBL3343366	CHEMBL279
CHEMBL2030438	CHEMBL4005
CHEMBL328029	CHEMBL3650
CHEMBL2386972	CHEMBL4005
CHEMBL2323141	CHEMBL2916
CHEMBL1915448	CHEMBL1974
CHEMBL377771	CHEMBL4282
CHEMBL1795619	CHEMBL5747
CHEMBL3962203	CHEMBL279
CHEMBL576982	CHEMBL279
CHEMBL4164843	CHEMBL3717
CHEMBL3596879	CHEMBL279
CHEMBL3915857	CHEMBL2148
CHEMBL1946262	CHEMBL4005
CHEMBL101464	CHEMBL279
CHEMBL233454	CHEMBL279
CHEMBL3775086	CHEMBL279
CHEMBL4078141	CHEMBL1974
CHEMBL112914	CHEMBL3650
CHEMBL3577116	CHEMBL279
CHEMBL3114771	CHEMBL2148
CHEMBL3687223	CHEMBL5568
CHEMBL4281394	CHEMBL5568
CHEMBL4166436	CHEMBL4247
CHEMBL4078075	CHEMBL1974
CHEMBL3814951	CHEMBL2148
CHEMBL3904441	CHEMBL1974
CHEMBL3703988	CHEMBL3717
CHEMBL4161643	CHEMBL5568
CHEMBL4211826	CHEMBL2815
CHEMBL2312304	CHEMBL3717
CHEMBL2332844	CHEMBL1974
CHEMBL564940	CHEMBL2148
CHEMBL205514	CHEMBL4282
CHEMBL1681958	CHEMBL279
CHEMBL1762781	CHEMBL4005
CHEMBL598852	CHEMBL3650
CHEMBL380944	CHEMBL279

CHEMBL4244708	CHEMBL1974
CHEMBL245966	CHEMBL279
CHEMBL2151932	CHEMBL4005
CHEMBL1090598	CHEMBL4005
CHEMBL1822974	CHEMBL3717
CHEMBL1796242	CHEMBL4247
CHEMBL1940275	CHEMBL279
CHEMBL1823254	CHEMBL3717
CHEMBL3747077	CHEMBL2148
CHEMBL4112318	CHEMBL4005
CHEMBL403661	CHEMBL1974
CHEMBL3814934	CHEMBL2148
CHEMBL3941123	CHEMBL4247
CHEMBL3329651	CHEMBL1974
CHEMBL3931691	CHEMBL279
CHEMBL516640	CHEMBL3956
CHEMBL147761	CHEMBL279
CHEMBL1644558	CHEMBL4005
CHEMBL3958497	CHEMBL1936
CHEMBL3416150	CHEMBL1974
CHEMBL4094670	CHEMBL2041
CHEMBL3586401	CHEMBL4005
CHEMBL4171749	CHEMBL5747
CHEMBL3937128	CHEMBL3717
CHEMBL3398175	CHEMBL4247
CHEMBL598434	CHEMBL279
CHEMBL1241679	CHEMBL279
CHEMBL3642425	CHEMBL2148
CHEMBL55594	CHEMBL3650
CHEMBL3673444	CHEMBL2815
CHEMBL206410	CHEMBL3650
CHEMBL4063976	CHEMBL3650
CHEMBL106571	CHEMBL2148
CHEMBL4209753	CHEMBL1974
CHEMBL2322769	CHEMBL4005
CHEMBL2322773	CHEMBL4005
CHEMBL1202478	CHEMBL3650
CHEMBL469025	CHEMBL279
CHEMBL3642334	CHEMBL2148
CHEMBL2057368	CHEMBL4005
CHEMBL2151323	CHEMBL2815
CHEMBL3900059	CHEMBL279
CHEMBL3654300	CHEMBL5747

CHEMBL1242033	CHEMBL4005
CHEMBL3673470	CHEMBL2815
CHEMBL1165499	CHEMBL4247
CHEMBL4114735	CHEMBL3717
CHEMBL2035028	CHEMBL4282
CHEMBL3335371	CHEMBL1936
CHEMBL155151	CHEMBL2916
CHEMBL3290305	CHEMBL4005
CHEMBL1077842	CHEMBL2148
CHEMBL1773601	CHEMBL4282
CHEMBL1258331	CHEMBL4282
CHEMBL240336	CHEMBL4005
CHEMBL1240566	CHEMBL279
CHEMBL3693558	CHEMBL4282
CHEMBL3890788	CHEMBL2148
CHEMBL3753157	CHEMBL279
CHEMBL524708	CHEMBL279
CHEMBL2012699	CHEMBL4282
CHEMBL1773581	CHEMBL4282
CHEMBL4163608	CHEMBL4282
CHEMBL1091714	CHEMBL4005
CHEMBL2158227	CHEMBL1936
CHEMBL252856	CHEMBL4282
CHEMBL1689442	CHEMBL2916
CHEMBL1290744	CHEMBL4005
CHEMBL400057	CHEMBL1936
CHEMBL2442139	CHEMBL3717
CHEMBL209774	CHEMBL1974
CHEMBL3813725	CHEMBL4005
CHEMBL3353355	CHEMBL279
CHEMBL3218849	CHEMBL4247
CHEMBL3770633	CHEMBL4005
CHEMBL4249784	CHEMBL2148
CHEMBL3896249	CHEMBL279
CHEMBL92516	CHEMBL279
CHEMBL199723	CHEMBL1974
CHEMBL1277620	CHEMBL2041
CHEMBL527066	CHEMBL3717
CHEMBL264382	CHEMBL279
CHEMBL3672558	CHEMBL4282
CHEMBL3977135	CHEMBL4005
CHEMBL255902	CHEMBL279
CHEMBL219886	CHEMBL279

CHEMBL4284396	CHEMBL2148
CHEMBL3938428	CHEMBL2815
CHEMBL2418949	CHEMBL4005
CHEMBL2017973	CHEMBL4005
CHEMBL3941879	CHEMBL2148
CHEMBL1241950	CHEMBL4005
CHEMBL4175060	CHEMBL5747
CHEMBL1204020	CHEMBL2916
CHEMBL594217	CHEMBL4005
CHEMBL2064508	CHEMBL4005
CHEMBL4114743	CHEMBL3717
CHEMBL2152254	CHEMBL4005
CHEMBL3904401	CHEMBL4005
CHEMBL226331	CHEMBL4282
CHEMBL3668018	CHEMBL279
CHEMBL1940275	CHEMBL1974
CHEMBL3667416	CHEMBL4005
CHEMBL1762782	CHEMBL4005
CHEMBL2323145	CHEMBL2916
CHEMBL4167072	CHEMBL3717
CHEMBL2086748	CHEMBL279
CHEMBL365585	CHEMBL279
CHEMBL2312652	CHEMBL3650
CHEMBL4074115	CHEMBL4247
CHEMBL3897417	CHEMBL2148
CHEMBL4241638	CHEMBL279
CHEMBL3648080	CHEMBL279
CHEMBL271131	CHEMBL279
CHEMBL1242844	CHEMBL4005
CHEMBL3891894	CHEMBL279
CHEMBL3952048	CHEMBL279
CHEMBL3347489	CHEMBL2916
CHEMBL1770494	CHEMBL2916
CHEMBL1957870	CHEMBL4005
CHEMBL3221125	CHEMBL3717
CHEMBL154216	CHEMBL2916
CHEMBL1170702	CHEMBL2916
CHEMBL2071451	CHEMBL3717
CHEMBL4067036	CHEMBL3717
CHEMBL3980022	CHEMBL279
CHEMBL3972478	CHEMBL3797
CHEMBL3589738	CHEMBL279
CHEMBL216703	CHEMBL279

CHEMBL3402763	CHEMBL3717
CHEMBL188978	CHEMBL279
CHEMBL218439	CHEMBL2148
CHEMBL589864	CHEMBL4005
CHEMBL3754301	CHEMBL279
CHEMBL3952637	CHEMBL4247
CHEMBL595373	CHEMBL279
CHEMBL516639	CHEMBL279
CHEMBL3736182	CHEMBL4247
CHEMBL2387079	CHEMBL4005
CHEMBL388978	CHEMBL1974
CHEMBL2064492	CHEMBL4005
CHEMBL1822798	CHEMBL3717
CHEMBL2381381	CHEMBL4005
CHEMBL3426917	CHEMBL1955
CHEMBL1173697	CHEMBL4282
CHEMBL1644571	CHEMBL4005
CHEMBL566010	CHEMBL3717
CHEMBL1823651	CHEMBL4282
CHEMBL3582356	CHEMBL4005
CHEMBL3655869	CHEMBL4005
CHEMBL3398166	CHEMBL4247
CHEMBL3423384	CHEMBL2916
CHEMBL1615189	CHEMBL4005
CHEMBL3656350	CHEMBL1974
CHEMBL3593764	CHEMBL2148
CHEMBL1242661	CHEMBL4005
CHEMBL597592	CHEMBL2041
CHEMBL3695624	CHEMBL4005
CHEMBL3932688	CHEMBL4282
CHEMBL1088857	CHEMBL2148
CHEMBL3600770	CHEMBL4005
CHEMBL3394477	CHEMBL3717
CHEMBL1645108	CHEMBL4005
CHEMBL4218103	CHEMBL2815
CHEMBL3674504	CHEMBL3717
CHEMBL4164803	CHEMBL5747
CHEMBL1807717	CHEMBL2041
CHEMBL3334568	CHEMBL3717
CHEMBL13354	CHEMBL3650
CHEMBL1630542	CHEMBL4005
CHEMBL3115500	CHEMBL4247
CHEMBL4214476	CHEMBL279

CHEMBL2088256	CHEMBL3650
CHEMBL3653155	CHEMBL4005
CHEMBL3354188	CHEMBL279
CHEMBL3746341	CHEMBL2148
CHEMBL156248	CHEMBL2916
CHEMBL462707	CHEMBL3717
CHEMBL3883989	CHEMBL4005
CHEMBL2032035	CHEMBL3717
CHEMBL3334553	CHEMBL3717
CHEMBL3950207	CHEMBL3717
CHEMBL1081852	CHEMBL4282
CHEMBL2312649	CHEMBL4282
CHEMBL1083305	CHEMBL4005
CHEMBL3690510	CHEMBL279
CHEMBL2103868	CHEMBL3717
CHEMBL1822801	CHEMBL3717
CHEMBL3925815	CHEMBL2148
CHEMBL1241389	CHEMBL4005
CHEMBL3290148	CHEMBL2148
CHEMBL429410	CHEMBL279
CHEMBL3671823	CHEMBL2148
CHEMBL3577117	CHEMBL279
CHEMBL3218861	CHEMBL4247
CHEMBL2171124	CHEMBL1936
CHEMBL529663	CHEMBL1974
CHEMBL493911	CHEMBL4282
CHEMBL2037224	CHEMBL2815
CHEMBL3645547	CHEMBL2148
CHEMBL176078	CHEMBL279
CHEMBL3687195	CHEMBL4247
CHEMBL3098318	CHEMBL279
CHEMBL498769	CHEMBL3717
CHEMBL2087482	CHEMBL4005
CHEMBL4204150	CHEMBL3717
CHEMBL4284541	CHEMBL1974
CHEMBL4165354	CHEMBL3717
CHEMBL1915640	CHEMBL1974
CHEMBL3948901	CHEMBL4005
CHEMBL383030	CHEMBL279
CHEMBL524057	CHEMBL279
CHEMBL3667472	CHEMBL2148
CHEMBL3134602	CHEMBL4005
CHEMBL3899361	CHEMBL4005

CHEMBL485215	CHEMBL4282
CHEMBL3600686	CHEMBL4005
CHEMBL2071276	CHEMBL279
CHEMBL462338	CHEMBL279
CHEMBL4082186	CHEMBL279
CHEMBL3128062	CHEMBL4247
CHEMBL4248386	CHEMBL2148
CHEMBL98350	CHEMBL4005
CHEMBL3309962	CHEMBL3717
CHEMBL3673459	CHEMBL2815
CHEMBL3793268	CHEMBL4005
CHEMBL3959170	CHEMBL4247
CHEMBL4103824	CHEMBL5747
CHEMBL470192	CHEMBL4282
CHEMBL3813701	CHEMBL5747
CHEMBL2442129	CHEMBL3717
CHEMBL3355074	CHEMBL3797
CHEMBL3221129	CHEMBL3717
CHEMBL379372	CHEMBL279
CHEMBL3601923	CHEMBL4005
CHEMBL2448066	CHEMBL279
CHEMBL3113144	CHEMBL4282
CHEMBL46785	CHEMBL279
CHEMBL2409780	CHEMBL279
CHEMBL398989	CHEMBL279
CHEMBL270847	CHEMBL4282
CHEMBL2323783	CHEMBL3717
CHEMBL3645022	CHEMBL2148
CHEMBL3814770	CHEMBL4005
CHEMBL4097807	CHEMBL2916
CHEMBL3943168	CHEMBL3650
CHEMBL3687205	CHEMBL2041
CHEMBL3981577	CHEMBL279
CHEMBL3967044	CHEMBL4247
CHEMBL245948	CHEMBL279
CHEMBL3600784	CHEMBL4005
CHEMBL3644972	CHEMBL2148
CHEMBL3639478	CHEMBL4005

Supplementary Table S5. Physicochemical properties of the drug candidate compounds and drugs (lenvatinib, regorafenib, and sorafenib).

Drug candidate compounds	CHEMBL388978	CHEMBL1615189	CHEMBL328029	CHEMBL1165499	CHEMBL1773581	CHEMBL1773601	Lenvatinib	Regorafenib	Sorafenib
**SMILES**	CN[C@@H]1C[C@H]2O[C@]([C@@H]1OC)(C)n1c3ccccc3c3c1c1n2c2ccccc2c1c1c3CNC1=O	CC(=O)Nc1nc2c(s1)cc(cc2)c1cnc(c(c1)NS(=O)(=O)c1ccc(cc1)F)Cl	O=C1Nc2c(/C/1=C/c1cc3c([nH]1)CCCC3)cccc2	CN1CCN(CC1)c1ccc(cn1)c1cnc2c(c1)N(CCN2)Cc1cc(F)ccc1F	CC(=O)Nc1nc2c(s1)cc(cc2)c1ccnc(n1)NS(=O)(=O)c1ccc(cc1)C	COc1ccc(cc1)S(=O)(=O)Nc1cccc(c1)c1ccc2c(c1)sc(n2)NC(=O)C	COc1cc2nccc(c2cc1C(=O)N)Oc1ccc(c(c1)Cl)NC(=O)NC1CC1	CNC(=O)c1nccc(c1)Oc1ccc(c(c1)F)NC(=O)Nc1ccc(c(c1)C(F)(F)F)Cl	CNC(=O)c1nccc(c1)Oc1ccc(cc1)NC(=O)Nc1ccc(c(c1)C(F)(F)F)Cl
**Formula**	C28H26N4O3	C20H14ClFN4O3S2	C17H16N2O	C24H26F2N6	C20H17N5O3S2	C22H19N3O4S2	C21H19ClN4O4	C21H15ClF4N4O3	C21H16ClF3N4O3
**Molecular weight**	466.53 g/mol	476.93 g/mol	264.32 g/mol	436.50 g/mol	439.51 g/mol	453.53 g/mol	426.85 g/mol	482.82 g/mol	464.82 g/mol
**Num. heavy atoms**	35	31	20	32	30	31	30	33	32
**Num. arom. heavy atoms**	20	21	11	18	21	21	16	18	18
**Fraction Csp3**	0.32	0.05	0.24	0.33	0.10	0.09	0.19	0.10	0.10
**Num. rotatable bonds**	2	6	1	4	6	7	8	9	9
**Num. H-bond acceptors**	4	6	1	5	6	5	5	8	7
**Num. H-bond donors**	2	2	2	1	2	2	3	3	3
**Molar Refractivity**	139.39	119.25	83.92	135.40	117.04	122.97	112.86	112.44	112.48
**TPSA**	69.45 Å^2	137.67 Å^2	44.89 Å^2	47.53 Å^2	150.56 Å^2	134.01 Å^2	115.57 Å^2	92.35 Å^2	92.35 Å^2

Supplementary Table S6. Lipophilicity of the drug candidate compounds and drugs (lenvatinib, regorafenib, and sorafenib).

**Drug candidate compounds**	CHEMBL388978	CHEMBL1615189	CHEMBL328029	CHEMBL1165499	CHEMBL1773581	CHEMBL1773601	Lenvatinib	Regorafenib	Sorafenib
**Log Po/w (iLOGP)**	3.19	2.50	2.51	3.29	1.60	2.47	2.68	3.51	3.42
**Log Po/w (XLOGP3)**	3.24	4.03	2.95	3.59	3.05	4.01	2.75	4.17	4.07
**Log Po/w (WLOGP)**	3.39	6.03	2.71	2.58	4.52	5.43	3.82	6.88	6.32
**Log Po/w (MLOGP)**	2.60	2.54	2.44	3.09	1.29	2.38	2.10	3.28	2.91
**Log Po/w (SILICOS-IT)**	3.02	4.34	4.34	3.51	3.23	3.90	2.70	4.21	3.78
**Consensus Log Po/w**	3.09	3.89	2.99	3.21	2.74	3.64	2.81	4.41	4.10

Supplementary Table S7. Water solubility of the drug candidate compounds and drugs (lenvatinib, regorafenib, and sorafenib).

**Drug candidate compounds**	CHEMBL388978	CHEMBL1615189	CHEMBL328029	CHEMBL1165499	CHEMBL1773581	CHEMBL1773601	Lenvatinib	Regorafenib	Sorafenib
**Log *S* (ESOL)**	-5.06	-5.44	-3.68	-4.96	-4.61	-5.22	-4.09	-5.27	-5.11
**Solubility**	4.02e-03 mg/ml ; 8.62e-06 mol/l	1.73e-03 mg/ml ; 3.62e-06 mol/l	5.54e-02 mg/ml ; 2.10e-04 mol/l	4.78e-03 mg/ml ; 1.10e-05 mol/	1.08e-02 mg/ml ; 2.46e-05 mol/l	2.75e-03 mg/ml ; 6.06e-06 mol/l	3.50e-02 mg/ml ; 8.21e-05 mol/l	2.59e-03 mg/ml ; 5.37e-06 mol/l	3.62e-03 mg/ml ; 7.79e-06 mol/l
**Class**	Moderately soluble	Moderately soluble	Soluble	Moderately soluble	Moderately soluble	Moderately soluble	Moderately soluble	Moderately soluble	Moderately soluble
**Log *S* (Ali)**	-4.37	-6.62	-3.56	-4.27	-5.88	-6.53	-4.83	-5.82	-5.71
**Solubility**	1.98e-02 mg/ml ; 4.25e-05 mol/l	1.13e-04 mg/ml ; 2.38e-07 mol/l	7.36e-02 mg/ml ; 2.79e-04 mol/l	2.32e-02 mg/ml ; 5.31e-05 mol/l	5.82e-04 mg/ml ; 1.32e-06 mol/l	1.35e-04 mg/ml ; 2.97e-07 mol/l	6.29e-03 mg/ml ; 1.47e-05 mol/l	7.35e-04 mg/ml ; 1.52e-06 mol/l	8.98e-04 mg/ml ; 1.93e-06 mol/l
**Class**	Moderately soluble	Poorly soluble	Soluble	Moderately soluble	Moderately soluble	Poorly soluble	Moderately soluble	Moderately soluble	Moderately soluble
**Log *S* (SILICOS-IT)**	-7.59	-8.74	-5.76	-7.65	-7.90	-8.37	-6.84	-8.86	-8.60
**Solubility**	1.19e-05 mg/ml ; 2.56e-08 mol/l	8.70e-07 mg/ml ; 1.82e-09 mol/l	4.63e-04 mg/ml ; 1.75e-06 mol/l	9.86e-06 mg/ml ; 2.26e-08 mol/l	5.57e-06 mg/ml ; 1.27e-08 mol/l	1.94e-06 mg/ml ; 4.29e-09 mol/l	6.16e-05 mg/ml ; 1.44e-07 mol/l	6.63e-07 mg/ml ; 1.37e-09 mol/l	1.16e-06 mg/ml ; 2.50e-09 mol/l
**Class**	Poorly soluble	Poorly soluble	Moderately soluble	Poorly soluble	Poorly soluble	Poorly soluble	Poorly soluble	Poorly soluble	Poorly soluble

Supplementary Table S8. Medicinal chemistry of the drug candidate compounds and drugs (lenvatinib, regorafenib, and sorafenib).

Drug candidate compounds	CHEMBL388978	CHEMBL1615189	CHEMBL328029	CHEMBL1165499	CHEMBL1773581	CHEMBL1773601	Lenvatinib	Regorafenib	Sorafenib
**PAINS**	0 alert	0 alert	0 alert	0 alert	0 alert	0 alert	0 alert	0 alert	0 alert
**Brenk**	0 alert	1 alert: 2-halo_pyridine	1 alert: michael_acceptor_1	0 alert	0 alert	0 alert	0 alert	0 alert	0 alert
**Leadlikeness**	No; 1 violation: MW>350	No; 2 violations: MW>350, XLOGP3>3.5	Yes	No; 2 violations: MW>350, XLOGP3>3.5	No; 1 violation: MW>350	No; 2 violations: MW>350, XLOGP3>3.5	No; 2 violations: MW>350, Rotors>7	No; 3 violations: MW>350, Rotors>7, XLOGP3>3.5	No; 3 violations: MW>350, Rotors>7, XLOGP3>3.5
**Synthetic accessibility**	4.93	3.37	2.82	3.62	3.31	3.30	2.88	3.04	2.87
